# A self-assembled bilayer polypeptide-engineered hydrogel for spatiotemporal modulation of bactericidal and anti-inflammation process in osteomyelitis treatment

**DOI:** 10.1186/s12951-022-01614-3

**Published:** 2022-09-15

**Authors:** Xiaoting Xie, Jiemao Wei, Bin Zhang, Wei Xiong, Zhiyi He, Yayun Zhang, Chenghao Gao, Yuandi Zhao, Bo Liu

**Affiliations:** 1 Britton Chance Center for Biomedical Photonics at Wuhan National Laboratory for Optoelectronics-Hubei Bioinformatics & Molecular Imaging Key Laboratory, Department of Biomedical Engineering, College of Life Science and Technology, Huazhong University of Science and Technology, Wuhan, 430074 Hubei People’s Republic of China; 2 Department of Orthopedic Surgery, Tongji Hospital, Tongji Medical College, Huazhong University of Science & Technology, 1095 Jiefang Avenue, Wuhan, 430030 Hubei People’s Republic of China; 3 Key Laboratory of Biomedical Photonics (HUST), Ministry of Education, Huazhong University of Science and Technology, Wuhan, 430074 Hubei People’s Republic of China

**Keywords:** Engineered polypeptide hydrogel, Antibacterial, Spatiotemporal regulation, Bone repairing, Osteomyelitis

## Abstract

**Background:**

Drug resistance of pathogens and immunosuppression are the main causes of clinical stagnation of osteomyelitis. The ideal treatment strategy for osteomyelitis is to achieve both efficient antibacterial and bone healing through spatiotemporal modulation of immune microenvironment.

**Methods:**

In this study, a bilayer hydrogel based on genetically engineered polypeptide AC_10_A and AC_10_ARGD was prepared by self-assembly. Ag_2_S QDs@DSPE-mPEG_2000_-Ce6/Aptamer (AD-Ce6/Apt) was loaded in the top layer AC_10_A hydrogel (AA) for antibacterial, and bone marrow-derived mesenchymal stem cells (BMSCs) were loaded in the lower layer AC_10_ARGD hydrogel (MAR) for bone healing. The AD-Ce6/Apt can be released from the AA hydrogel to target *S. aureus* before bacterial biofilm formation and achieved significant bactericidal effect under irradiation with a 660 nm laser. Moreover, AD-Ce6/Apt can induce M1 type polarization of macrophages to activate the immune system and eliminate residual bacteria. Subsequently, BMSCs released from the MAR hydrogel can differentiate into osteoblasts and promote the formation of an anti-inflammatory microenvironment by regulating the M2 type polarization of macrophages. The bilayer AA-MAR hydrogel possessed good biocompatibility.

**Results:**

The in vitro and in vivo results showed that the AA-MAR hydrogel not only realized efficient photodynamic therapy of *S. aureus* infection, but also promoted the transformation of immune microenvironment to fulfill the different needs of each stage, which ultimately improved bone regeneration and mechanical properties post-surgery.

**Conclusion:**

This work presents an approach for spatiotemporal modulation of immune microenvironment in the treatment of osteomyelitis.

**Graphical Abstract:**

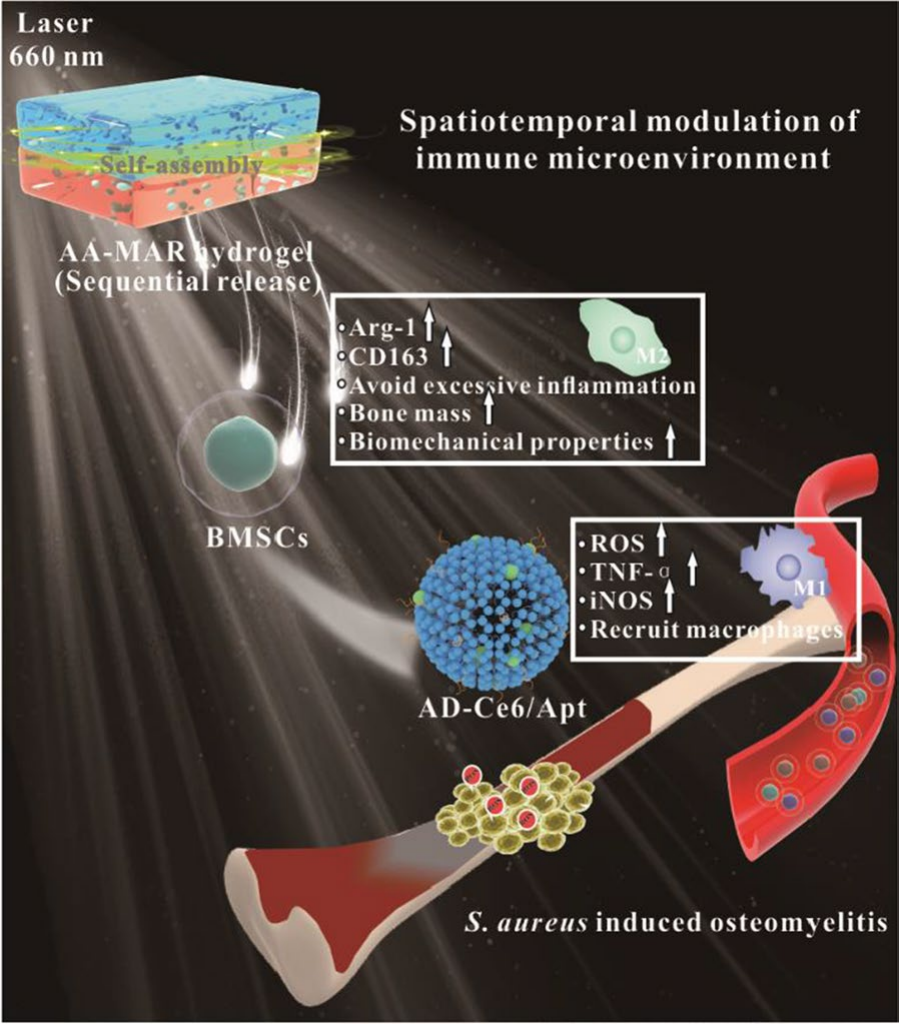

**Supplementary Information:**

The online version contains supplementary material available at 10.1186/s12951-022-01614-3.

## Introduction

Osteomyelitis caused by infecting microorganisms is a major clinical challenge, resulting in a huge medical and financial burden due to the rapid emergence of drug-resistant bacteria [1–3]. Gram-positive *Staphylococcus aureus* (*S. aureus*) derived from blood or external contact can cause severe inflammation, local bone loss and destruction, blood vessel damage, and other life-threatening diseases [4–6]. The efficiency of treatment can be greatly improved if *S. aureus*-induced osteomyelitis can be eliminated early in vivo and the damaged bone immune microenvironment can be subsequently remodeled. Photodynamic therapy (PDT) based on activated photosensitizers (PS) is emerging as a powerful antimicrobial method in vivo because it can avoid the disadvantages of drug resistance, bacteria residue, and weak bone mechanics caused by antibiotic administration and multiple surgical debridement [7–9]. High levels of reactive oxygen species (ROS) generated by PS can induce oxidative damage of biomacromolecules (proteins, lipids, and DNA) and metabolic dysfunction of bacteria [10, 11]. Moreover, the extracellular ROS generated by PS can induce macrophages to differentiate into M1 type and remove the immunosuppression caused by biofilms, thus enhancing the bactericidal effect [12, 13]. Although some PS have been designed for the treatment of osteomyelitis, they have only focused on the first stage (anti-bacteria) and ignored the dynamic healing process, which has become a major obstacle to clinical application. Therefore, there is an urgent need to develop an alternative strategy for sequential treatment of osteomyelitis.

A major challenge is how to spatially and temporally regulate the transition of the different healing stages. Hydrogels are a class of polymer network formed by physical or chemical cross-linking, which are suitable for the growth of surrounding tissues and cells and reduce the toxicity to organisms [14–16]. Recent advances in loading drugs inside hydrogel networks provide diverse opportunities to address osteomyelitis [17, 18]. These hydrogels contain one or more functions, such as good antibacterial properties, superior mechanical properties and strong tissue adhesion, depending on the therapeutic need [19, 20]. Whlie multifunctional monolayer hydrogels are difficult to meet different needs at each healing stage. Notably, with the development of genetic engineering technology, physical multilayer hydrogels are more conductive to be designed from scratch to achieve sequential regulation. Engineered polypeptide-based hydrogel with biological motifs have attracted much attention due to excellent biocompatibility and tunability. Engineered polypeptide-based materials (such as mussel glue protein, elastin-like polypeptide, filament protein, silk fibroin, coiled loop domain, leucine zipper peptide, and β-hairpin peptide) interact through non-covalent bonds (e.g., hydrogen bond, π–π, electrostatic interaction, and van der Waals interaction) to form a dynamic and reversible hydrogel system [21, 22]. Different polypeptide-based hydrogels can form heterogeneous multilayer hydrogels, which can effectively sequential release required drugs in each stage and spatiotemporal regulates the transition of different healing stages at the same time. However, few heterogeneous multilayer hydrogels formed with engineered polypeptides have been developed for spatiotemporal regulation.

**Fig. 1:**
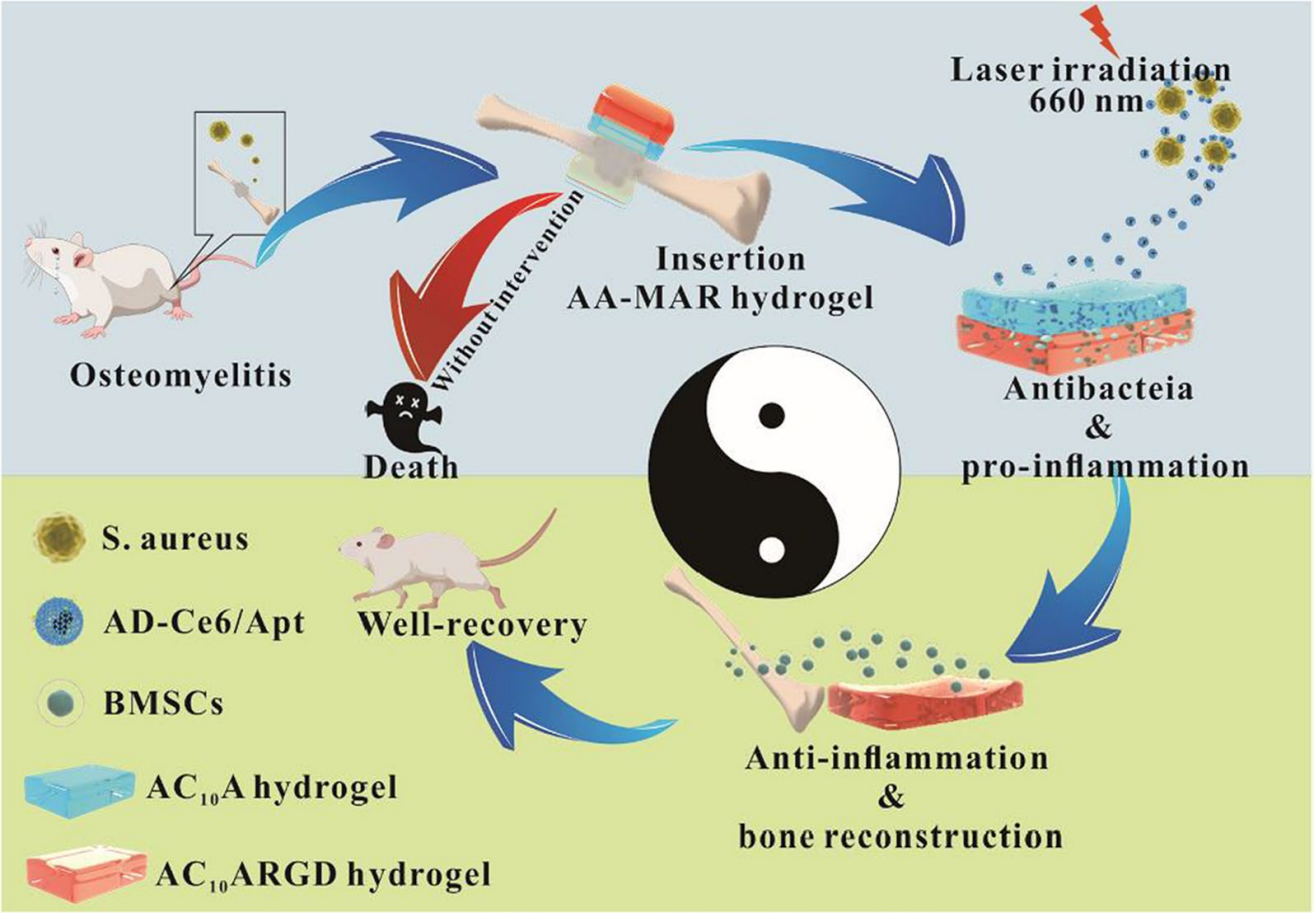
Schematic illustration of the bilayer AA-MAR hydrogel for spatiotemporal modulation of bactericidal and anti-inflammation process in osteomyelitis treatment

Herein, a bilayer multifunctional hydrogel based on engineered polypeptides AC_10_A and AC_10_ARGD was prepared for spatiotemporal modulation of immune microenvironment during osteomyelitis treatment (Fig. 1). Ag_2_S QDs@DSPE-mPEG_2000_-Ce6/Aptamer (AD-Ce6/Apt) was loaded in the top layer AC_10_A hydrogel (AA), and bone marrow-derived mesenchymal stem cells (BMSCs) were loaded in the lower layer AC_10_ARGD hydrogel (MAR). The antibacterial AA hydrogel and the bone repair-promoting MAR hydrogel can form a bilayer hydrogel (AA-MAR) by self-assembly. The AD-Ce6/Apt was first released from AA hydrogel to targeted kill *S. aureus* through PDT in the early stage of healing process. Simultaneously extracellular ROS induced macrophages to M1 type polarization and formed pro-inflammatory immune microenvironment, which helping to remove residual bacteria. The fluorescence of Ag_2_S QDs was used to monitor the release of AA hydrogel before biofilm formation. BMSCs encapsulated in the lower MAR were subsequently released. On the one hand, BMSCs encapsulated in the MAR hydrogel can keep cell activity and differentiate to osteoblasts. On the other hand, BMSCs can mediate macrophages differentiation into M2 type to promote anti-inflammation immunoenvironment formation. The results showed that the AA-MAR hydrogel presented excellent biocompatibility and realized sequential release of bioactive structure to achieve antibacterial and bone remodeling orderly. Therefore, this work provides a new approach to osteomyelitis treatment, which not only realize efficient sterilization through photodynamic therapy, but also promote bone repair by spatiotemporal modulation of immune microenvironment.

## Experimental section

### Materials

NheI and SpeI were obtained from New England Biolabs. Nickel nitrilotiacetic acid resin was obtained from Qiagen. 1-dodecanethiol (DT, 98%), diethyldithiocarbamic acid silver salt (98%), trichlormethane, acetone, dimethyl sulfoxide (DMSO), ampicillin, and kanamycin were purchased from Sinopharm Group Chemical Reagent Co. Ltd. Chlorin e6 (Ce6) was obtained from Frontier Scientific Inc., and 1,2-Distearoyl-sn-glycero-3-phosphoethanolamine-N-[amine methoxy (polyethylene glycol)_2000_] (DSPE-mPEG_2000_-NH_2_) was purchased from Avanti. Ultrapure water (Millipore Mill-Q grade, 18.2 MΩ) was used in all the experiments. 1-(3-Dimethylaminopropyl)-3-ethylcarbodiimide hydrochloride (EDC) and N-Hydroxysuccinimide (NHS) were obtained from Shanghai Macklin Biochemical Co., Ltd (Shanghai, China). DMEM/F12 and RPMI 1640 were purchased from Thermo Fisher Scientific. *Staphylococcus aureus’* species-identifiable aptamer was obtained from Tsingke Biotechnology Co., Ltd. All reagents and solvents are of analytical grade and used without further purification.

### Preparation of DE-Ce6

Ce6 (7 mg) in a round-bottom flask was mixed with 2 mL of DMSO under vigorous stirring at room temperature (RT). After stirring for 15 min, 5 mg of DSPE-mPEG_2000_-NH_2_, 3 mg of dicyclohexylcarbodiimide, and 2 mL of 4-dimethylaminopyridine were added, and the mixture was stirred for 6 h. The mixture was dialyzed against ultrapure water to obtain DSPE-mPEG_2000_-Ce6 (DE-Ce6) [23].

### Preparation of AD-Ce6/Apt

Ag_2_S QDs was synthesized following previously published methods and dissolved in chloroform [24, 25]. DSPE-mPEG_2000_-NH_2_ (0.5 mg) were dissolved in 1 mL of chloroform, Ag_2_S QDs (500 μL, 1 mg\mL) and DE-Ce6 (750 μL, 1 mg\mL) were added and lightly stirred at room temperature. After stirring for 30 min, the solution was dried by a vacuum pump. Finally, 400 μL of PBS was added to obtain Ag_2_S QDs@DSPE-mPEG_2000_-Ce6 (AD-Ce6).

To prepare aptamer modified AD-Ce6, 1 nmol of *Staphylococcus aureus’* species-identifiable aptamer (5′-HOOC-GCTAACCCCCCCAGTCCGTCCTCCCAGCCTGACACACCGCCA-3′) was dissolved in 100 μL of ultrapure water, and 2.8 μg of EDC was added [26–28]. The pH of solution was adjusted to 4.5. NHS (3.2 μg) and 400 μL of AD-Ce6 were added, and the pH of the mixture was adjusted to 8. After stirring for 18 h, the mixture was centrifuged (8000*g*, 5 min) and suspended in 500 μL of PBS. The morphologies of AD-Ce6/Apt were characterized by a G20 U-Twin TEM (FEI, USA). Size distribution and zeta potential were measured by a ZS90 Zeta Sizer (Malvern, UK). The absorbance spectra were recorded using a UV 2500 UV–vis spectrophotometer (Shimadzu, Japan). The fluorescence spectra were characterized by a LS-55 spectrophotometer (PerkinElmer, USA).

### Isolation and culture of rat BMSCs

BMSCs was harvested from femurs and tibias of 4-week-old Sprague Dawley rats [29]. Bone marrow was extruded by penetrating an 18-gaugle needle into the medulla of the bone and flushed out with 5 mL of DMEM/F12. The obtained cells were centrifuged at 1000*g* for 10 min. The precipitation was re-suspended by 8 mL of DMEM/F12 and cultured at 37 °C, 5% CO_2_ for 24 h and the non-adherent cells were abandoned. Every other day, media were removed, and fresh media were added.

### Preparation of AA hydrogel and MAR hydrogel

PUC_18_AC_10_A plasmid was gifted from Prof. David Tirrell of the California Institute of Technology, Pasadena, CA. PUC_18_AC_10_A plasmid was digested with NheI and SpeI, and AC_10_A fragment was ligated in the NheI and SpeI restriction sites of pQE9 to yield pQE9AC_10_A plasmid. And pQE9AC_10_ARGD plasmid was constructed by inserting RGD fragment into the SpeI site of pQE9AC10A plasmid(Additional file 1: Fig. S1). The structures and purification methods of polypeptide AC_10_A and AC_10_ARGD were prepared according to our previously reported methods (Additional file 1: Fig. S2) [30].

150 mg of lyophilized AC_10_A was added in 1 mL of AD-Ce6/Apt solution (4 μg\mL). The pH of the mixture was adjusted to 7.4, and the AA hydrogel (15% w/v) was obtained. 150 mg of lyophilized AC_10_ARGD was added in 1 mL of DMEM/F12, and the pH of the mixture was adjusted to 7.4. BMSCs (10^6^ cells) were mixed in the 1 mL of AC_10_ARGD hydrogel to obtain MAR hydrogel (15% w/v). The morphologies of hydrogels were determined on a Nova Nano SEM 450 (FEI, USA). The MAR hydrogel was treated with live/dead staining for 30 min, and the fluorescence images were collected using an Olympus FLUOVIEW FV1000 confocal laser scanning microscope.

### Biocompatibility of MAR hydrogel

MTT assay was used to investigate cell viability [19]. BMSCs and MAR hydrogel were seeded in 96-well plates and cultured at 37 ℃, 5% CO_2_ for 3 days. The wells were divided into three groups: (I) PBS + BMSCs, (II) AD-Ce6/Apt + BMSCs, and (III) AD-Ce6/Apt + MAR hydrogel (five parallels per group). After culture at 37 °C, 5% CO_2_ for 3 days, the 200 μL of fresh medium containing 20 μL of 3-(4,5)-dimethylthiahiazo(-z-y1)-3,5-di-phenytetrazoliumromide (MTT, 5 mg/mL) was added to each well. Culture supernatant was replaced with 100 μL of dimethyl sulfoxide (DMSO) after incubation for 4 h. The absorbance value at 490 nm of each well was recorded by an ELX 808 IU microplate reader.

The alkaline phosphatase (ALP) expression of BMSCs inside MAR hydrogel was measured to investigate the osteogenic activity. BMSCs (10^4^ cells) or 10 μL of MAR hydrogel (10^4^ cells) was seeded in a 24-well plate (five parallels per group) and added with 2 mL of osteogenic differentiation medium. After culture for 14 days, alkaline phosphatase stain kit was used to stain samples. The stained samples were imaged by a BX53 light microscope (Olympus, Japan).

### Release kinetics of AA hydrogel and AA-MAR hSydrogel

200 μL of AA hydrogel was centrifuged to the bottom of a 1.5 mL EP tube. 1 mL of PBS was added on the AA hydrogel, and the tube was put into an incubator at RT and 37 °C. The top solution was collected to detect the concentration of Ce6 and replaced by a new solution at proper intervals [31]. The amount of Ce6 released from the AA hydrogel was measured using a UV 2500 UV–vis spectrophotometer.

To study the sequential release of AA-MAR hydrogel in vitro, AA-MAR hydrogel (100 μL AA/100 μL MAR) was centrifuged to the bottom of a 1.5 mL EP tube, and 1 mL of PBS was added on the AA-MAR hydrogel. The tube was put into an incubator at RT and 37 °C, and the release kinetics of the AA-MAR hydrogel were recorded at proper intervals.

### In vitro analysis of anti-infective effect

*Staphylococcus aureus* (ATCC 25923) were cultured in Luria–Bertani broth medium (LB) and harvested at the exponential growth phase prior to further experiment [32]. The concentration of *S. aureus* was monitored by measuring the optical density (OD) at a wavelength of 600 nm. Before anti-infection experiments, the OD_600_ values of *S. aureus* solution were diluted to 0.1 corresponding to the concentration of 10^8^ CFU\mL. 200 μL of bacteria suspension (10^7^ CFU\mL) was seeded into a 96-well cell culture plate, and the wells were divided into seven groups: (I) PBS; (II) Laser; (III) AC_10_A; (IV) AD-Ce6/Apt; (V) AA; (VI) AD-Ce6/Apt + Laser; and (VII) AA + Laser. The anti-infection performance was assessed by the spread plate method (SPM), and bacteria were cultured on the agar at 37 °C for 24 h. The antibacterial efficiency can be calculated based on the equation:

$${\text{Antibacterial efficiency}} \left( \% \right) = \frac{{A_{Ctr} {-} A_{Experiment} }}{{A_{Ctr} }} \times 100\%$$

In this equation, “A_Ctr_” represents count of bacterial colony in PBS groups.

50 μL of PBS, AA hydrogel, or AD-Ce6/Apt (4 μg\mL) was put in 24-well plates, and 1 mL of *S. aureus* suspension (10^7^ CFU) was added. After co-culturing at 37 °C for 8 h, the plates of Laser, AD-Ce6/Apt + Laser, and AA + Laser groups were irradiated with a 660 nm laser for 5 min. The bacteria suspensions were cultured at 37 °C for another 2 days. The bacteria were washed three times with PBS, and 500 μL of 1% (v/v) crystal violet was added in plates for 15 min. The images of crystal violet stained bacteria were recorded. After adding100 μL of methanol in each well, the absorbance of each well at 590 nm was measured.

### AA hydrogel for biofilm eradication

1 mL of *S. aureus* suspension (10^6^ CFU) was added into a 24-well plate and cultured at 37 °C for 2 days [33]. 50 μL of AA hydrogel was added in the well. After incubation for 8 h, samples were irradiated with a 660 nm laser for 5 min. The *S. aureus* suspension treated with PBS was used as a control. The 24-well plate was centrifuged (4000*g*, 10 min), and the supernatants were discarded. 100 μL of PBS was added into each well. The solution was suspended with ultrasonic treatment and diluted 100 times. 10 μL of suspension was evenly coated on the agar and cultured at 37 °C overnight. The morphology of biofilms was observed by an SEM after treated with PBS or AA hydrogel. The biofilms were treated with 4% Paraformaldehyde Fix Solution (PFS) for 30 min and washed three times with PBS. To visually observe the viability of bacteria, the biofilms after treating with PBS or AA hydrogel were stained with SYTO9 and PI [34]. The images were recorded on an inverted fluorescence microscope (IX71, Olympus, Japan).

### The chemokine-like function of AA hydrogel

Bone marrow-derived macrophages (BMDMs) were harvested from femurs and tibias of 4-week-old Sprague Dawley rats [35]. Bone marrow was extruded by penetrating an 18-gaugle needle into the medulla of the bone and flushed out with 5 mL of RPMI1640. The obtained suspension was centrifuged at 1000*g* for 10 min. The supernatant was abandoned, and the precipitate was re-suspended with 10 mL of RPMI1640 (include 10 ng/mL of macrophages colony-stimulating factor 1 (Procell)). The suspension was seeded in dishes and cultured at 37 °C, 5% CO_2_. The supernatant was transferred to a new dish after culturing for 24 h, and the medium was refreshed every other day.

The chemokine-like function of AA hydrogel was verified using a transwell migration test. BMDMs (5 × 10^4^ cells/well) were seeded into an 8 μm transwell. 50 μL of PBS, 50 μL of lipopolysaccharide (LPS, 100 ng/mL), or 50 μL of AA hydrogel was added into the lower clambers. After incubation at 37 °C for 24 h, BMDMs on the bottom of the transwell were stained with DAPI and imaged on an inverted fluorescence microscope (IX71, Olympus, Japan).

The phagocytic activity of BMDMs stimulated by 50 μL of PBS, 50 μL of LPS (100 ng\mL), or 50 μL of AA hydrogel was investigated, respectively. Briefly, BMDMs were co-cultured with PBS, LPS, or AA hydrogel at 37 °C for 24 h, and the medium was discarded. The 1 mL of RMPI-1640 containing 10^7^ CFU\mL of *S. aureus* was added. After incubation for 1 h, the suspension was replaced by 1 mL of fresh RMPI-1640 containing 200 μg\mL of gentamicin and cultured for another 1 h. BMDMs were washed three times with 1 mL of PBS, and 1% Triton X-100 was added. After incubation for 30 min, the solution was centrifuged at 4000*g* for 10 min, and the precipitate was washed three times with 1 mL of PBS. 10 μL of suspension was evenly coated on the agar and cultured at 37 °C overnight.

### The gene expression analysis of BMDMs differentiation in vitro

BMDMs were digested by trypsin and seeded in a 6-well plate at a concentration of 1 × 10^5^ cells/well. After culturing at 37 °C for 24 h, 10 μL of PBS, 50 μL of AA hydrogel, 50 μL of MAR hydrogel, and 100 μL AA-MAR hydrogel (50 μL/50 μL) were added into the wells. The morphology of BMDMs was recorded by an optical microscope (Olympus, Japan). BMDMs in 6-well plates were collected at indicated time points and treated with 1 mL of RNA isolater (Vazyme, China). A fifth volume of chloroform was added, and the solution was violent mixed. After standing at 4 °C for 5 min, the solution was centrifuged at 12,000*g* for 15 min. The supernatant was transferred to a new EP tube, and isovolume isopropanol was added. After standing at 4 °C for 10 min, the solution was centrifuged at 12,000*g* for 10 min. The supernatant was discarded, and 500 μL of 75% ethanol was added. The solution was centrifuged at 12,000*g* for 5 min. The supernatant was discarded, and the precipitate was dissolved with 20 μL of diethypyrocarbonate treated water (Biosharp). The obtained total RNA samples were converted into complementary DNA using HiScript@III All-in-one RT SuperMix Perfect for qPCR kit (Vazyme, China). RT-qPCR was performed using Taq Pro Universal SYBR qPCR Master Mix kit (Vazyme, China) and Applied Biosystems™ QuantStudio™ 3. The primers used in RT-qPCR were described in Additional file 1: Table S1. The GAPDH was used as the house keeping gene, and gene expression levels of TNF-α, iNOS, Arg-1, and CD163 were analyzed [35].

### In vivo anti-infection evaluation

The establishment of animal model was approved by Tongji Medical College of Huazhong University of Science and Technology. All rats’ right hind limbs were carefully shaved and disinfected. Lateral incision was made over the tibia, and the muscle was bluntly dissected to expose the tibia. The tibia was fractured at the diaphysis, and a sterile wire was inserted into the tibia shaft and retracted. After fractures, bacteria inoculum (5 × 10^5^ CFU in 5 μL of PBS) were delivered through the fracture site using a 25-gaugle needle into the medullary cavity (refer to the establishment of rat models of acute staphylococcal osteomyelitis) [25].

The fracture site was subsequently treated with 100 μL of PBS, AA-MAR hydrogel (25 μL/50 μL), AA-MAR hydrogel (50 μL/50 μL), AA-MAR hydrogel (100 μL/50 μL), AA-MAR hydrogel (150 μL/50 μL). The muscle and skin were sutured back into place, and saline solution was applied to clean skin. The fracture sites of the tibia in AA-MAR groups were irradiated with a 660 nm laser for 5 min (0.6 W cm^−2^) on day 1 and 3. For anti-infection evaluation, all rats were sacrificed using excessive doses of isofluorane. The tissues around infection sites, tibias, and needles were collected and immersed in 5 mL of PBS. Tissues and tibias were ground with a sterile mortar and uniformly dispersed by ultrasonic concussion [34]. The suspension was diluted 50,000 times. 10 μL of the diluted suspension was evenly coated on the agar and cultured at 37 °C for 24 h. Meanwhile, these tissues were fixed with 5 mL of 4% PFS and processed using Gram staining, and samples were observed and photographed by an optical microscope. To investigate the infection degree, the infected bone tissues were stained by Gram solution. The degradation of the AA-MAR hydrogel in vivo were measured by the fluorescence of Ag_2_S QDs in the AA hydrogel. The fluorescence image was collected on a home-built fluorescence system.

### In vivo immune responses assay

Blood samples of different groups were collected from the tail of *S. aureus* induced osteomyelitis rats after treatment for 1, 2, 3, 5, and 7 days and were centrifuged at 3000*g* for 15 min. The concentration of TNF-α and IL-10 in serum were assessed by Elisa kits according to the manufacturer’s protocol. In addition, the gene expression of TNF-α and IL-10 in tissue samples was analyzed by RT-qPCR referring to the method of in vitro assay. For more intuitive analysis of infiltrating immune cells of different groups, rats were sacrificed at 3 and 7 days after fractures. Tissue sections were stained with different antibodies: CD86, CD163, and DAPI. The slides were imaged on a confocal microscope (Fluoview 3000, Olympus, Japan). The corresponding H&E staining slides were also made up to present the infiltration of immune cells.

### Micro-CT and mechanical testing of tibia

After different treatments for five weeks, the rats were killed by excessive doses of isofluorane. The tibias were dissected, and the wires were removed. The tibias were placed in EP tubes soaked in saline until further experiments. Samples were scanned using a micro-CT (SkyScan1176, Bruker, China) to analyze fracture healing. Three-point bending test was approached by a universal testing machine (WDW-2, Jinan Hensgrand Instrument Co., Ltd.), and the maximum stress and bending stiffness of tibia after different treatments were recorded.

### Statistical analysis

All results in this work were expressed as mean values ± standard deviation with n ≥ 3. A one-way analysis of variance (ANOVA) and Student’s t test were performed for significance analysis. Statistical significance was considered when *p < 0.05, **p < 0.01, and ***p < 0.001.

## Results and disscussion

### Preparation and characterization of AD-Ce6/Apt

**Fig. 2:**
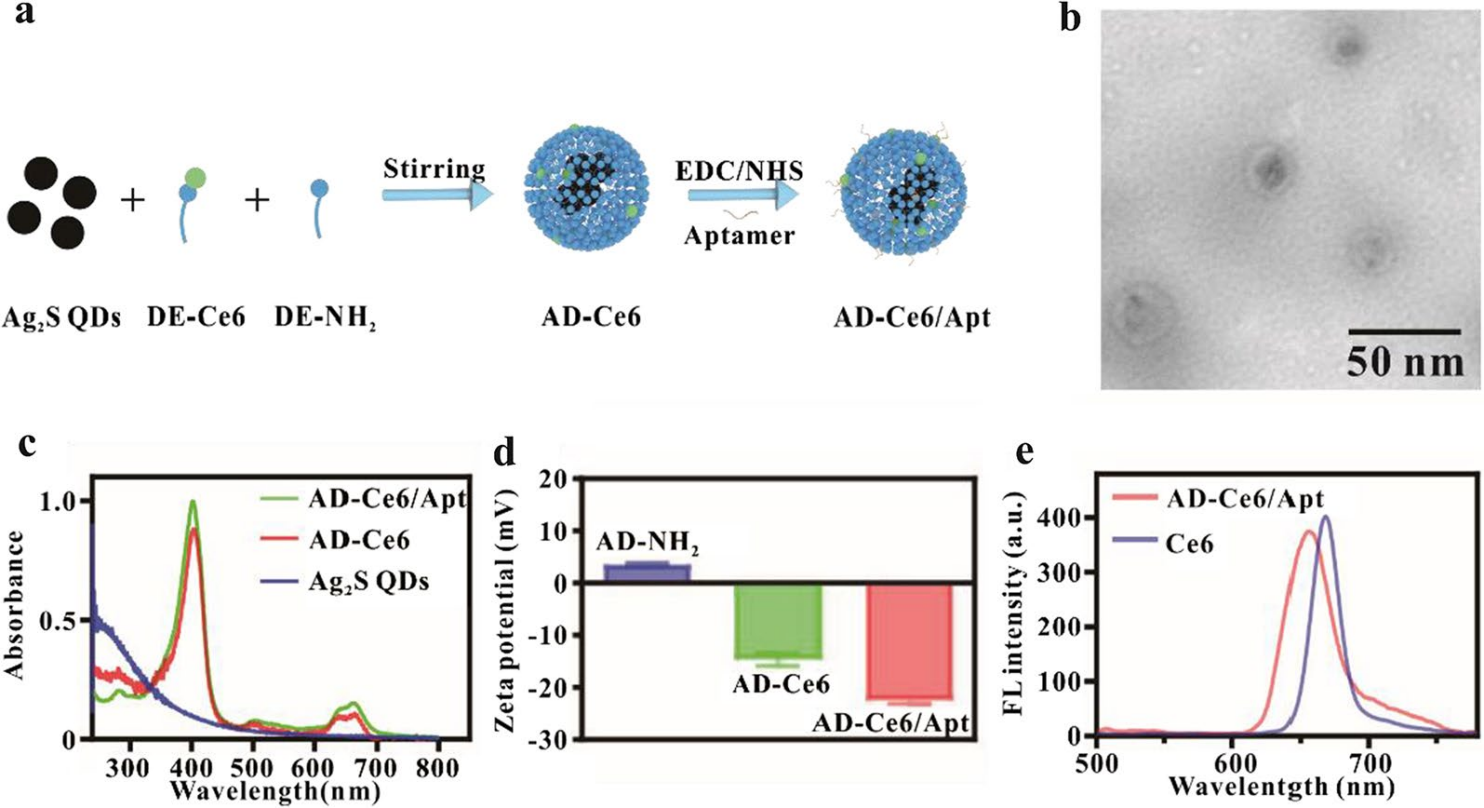
Characterization of AD-Ce6/Apt. **a** Schematic illustration of the synthesis of AD-Ce6/Apt. **b** TEM images of AD-Ce6/Apt. UV–vis-NIR absorbance spectra (**c**) and zeta potential (**d**) of AD-Ce6/Apt. **e** The fluorescence spectra of Ce6 and AD-Ce6/Apt (4 μg\mL)

Double-layer AA-MAR hydrogels were designed to eradicate bacteria and repair bone tissue in the treatment of osteomyelitis by sequential release. The top AA hydrogels loaded with AD-Ce6/Apt are mainly used for *S. aureus* eradication and immune microenvironment modulation. Three steps were taken to obtain the AD-Ce6/Apt. To synthesize DE-Ce6, the carboxyl of Ce6 was conjugated to amino of DEPC-mPEG_2000_-NH_2_ through EDC/NHS coupling chemistry. AD-Ce6 was prepared by self-assembly of DE-NH_2_, DE-Ce6, and Ag_2_S QDs. To enhance the *S. aureus*’ (main pathogen) targeting, the specific aptamer was introduced on the surface of AD-Ce6 particles through the covalent coupling method (Fig. 2a). Transmission electron microscope (TEM) images exhibited that the prepared AD-Ce6/Apt possessed a homogeneous spherical structure with average size 22.5 ± 2 nm (Fig. 2b). After Ag_2_S QDs self-assembled with DE-Ce6, the UV–vis absorption spectrum of AD-Ce6 presented the characteristic absorption peaks of Ce6 at 403 nm and 664 nm. And the UV–vis absorption spectrum of AD-Ce6/Apt presented the characteristic absorption peaks of aptamer at 260 nm and Ce6 at 403 nm and 664 nm. In addition, significant reverse of zeta potentials was observed from 3.55 to − 14.68 mV after modification with Ce6, and the zeta potential of AD-Ce6/Apt was further reduced after conjugating with aptamer (Fig. 2d). These results indicated that all components were integrated into the AD-Ce6/Apt, and the probe was successfully prepared.

Our previously reported results showed that the fluorescence of Ce6 was easy to be quenched by Ag_2_S QDs, and simultaneously the photodynamic performance of Ce6 was decreased [23]. To optimize the ratio of Ag_2_S QDs and Ce6 in the AD-Ce6/Apt, fluorescence spectra of the AD-Ce6/Apt with different ratios were measured. When the ratios of Ag_2_S QDs: Ce6 were 1:1, 2:1, and 4:1, the fluorescence of Ce6 was quenched by Ag_2_S QDs, indicating that the fluorescence quenching efficiency of Ce6 increased with the proportion of Ag_2_S QDs. To optimize the photodynamic performance of AD-Ce6/Apt, we down regulated the ratio of Ag_2_S QDs in the AD-Ce6/Apt. The result showed that the fluorescence of ce6 increased continuously with the decrease of Ag_2_S QDs. When the ratio of Ag_2_S QDs: Ce6 was regulated to 1:1.5, the negligible difference was exerted on the fluorescence intensity of free Ce6 and AD-Ce6/Apt at a constant concentration of Ce6 (Fig. 2e). Compared with the fluorescence emission peak of free Ce6, the slight blue-shift of the fluorescence emission peak of AD-Ce6/Apt was observed due to the linkage of phospholipid. These results suggested that the fluorescence of Ce6 was not obviously affected under the Ag_2_S QDs: Ce6 ratio of 1:1.5. Therefore, the Ag_2_S QDs and Ce6 ratio of 1:1.5 in the AD-Ce6/Apt was utilized for subsequent experiments.

### Morphology and structure characterization of AA, MAR, and AA-MAR hydrogel

**Fig. 3:**
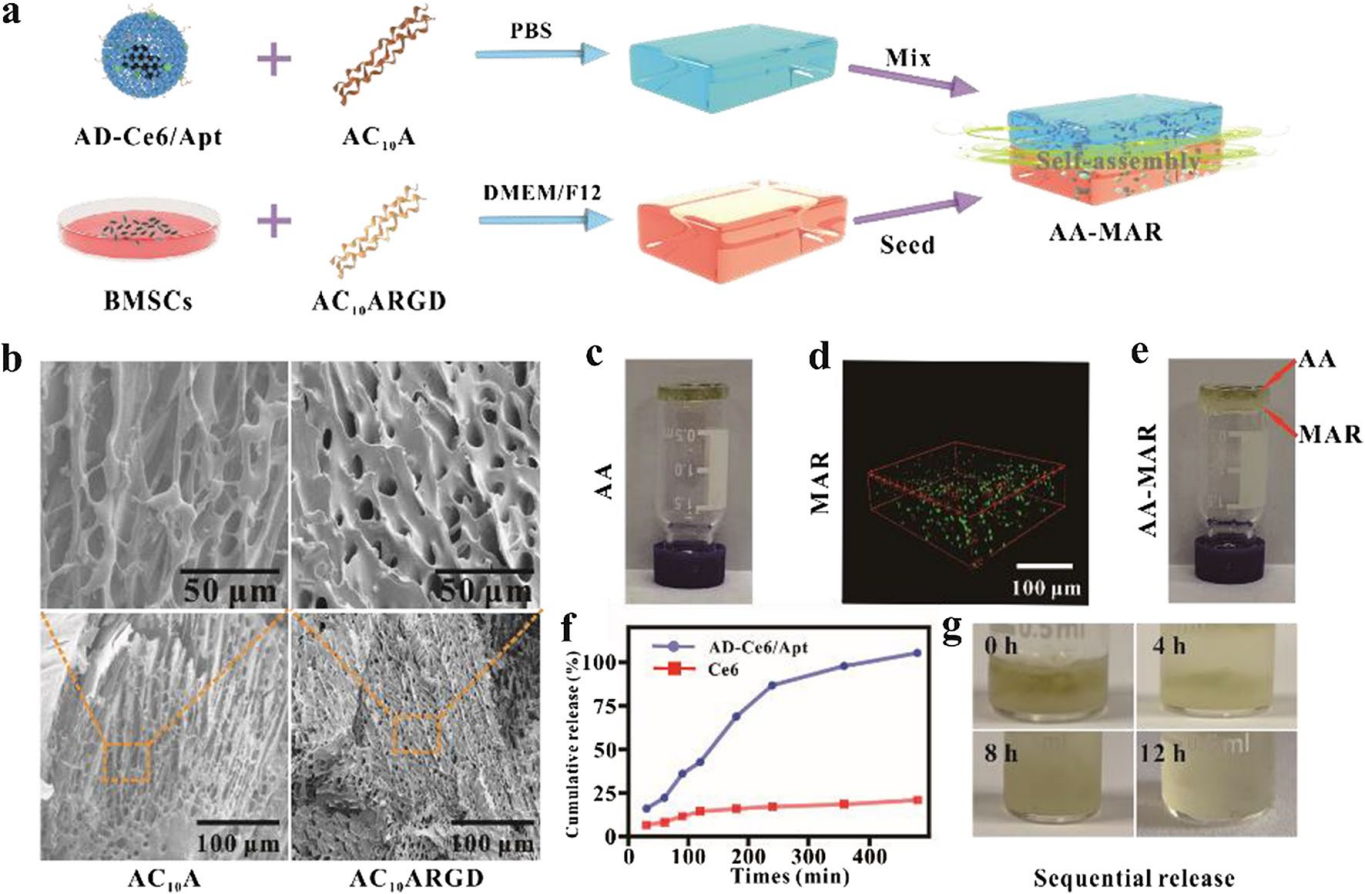
Characterization of AA-MAR hydrogel. **a** Schematic illustration of the preparation of AA-MAR hydrogel. **b** SEM images of AC_10_A hydrogel and AC_10_ARGD hydrogel. **c** Photograph of AA hydrogel. **d** 3D-reconstruction of fluorescence image of the live/dead stained BMSCs in the MAR hydrogel. **e** Photograph of self-assembly AA-MAR hydrogel. **f** The cumulative release of AD-Ce6/Apt from the AA hydrogel. **g** Photographs of sequential release of the AA-MAR hydrogel at 0, 4, 8, and 12 h

AC_10_A polypeptide is composed of a disordered water-soluble motif C_10_ connected to the leucine zipper-like motif A at both ends, and A domain can self-assemble to form a tetramer [36–38]. AC_10_A polypeptide can form hydrogel by self-assembly. The upper layer of AA-MAR hydrogel consisted of an AC_10_A hydrogel loaded with AD-Ce6/Apt. And the lower layer was composed of AC_10_ARGD hydrogel loaded with BMSCs. The introduction of RGD in the AC_10_A polypeptide can enhance the adhesion, stretching and differentiation of stem cells [39]. The AA hydrogel and MAR hydrogel are easy to form an integral bilayer hydrogel by self-assembly of A domain at the interface (Fig. 3a). The morphology of freeze-dried AC_10_A and AC_10_ARGD hydrogels was first observed by scanning electron microscope (SEM). The results of SEM exhibited that micro-sized pores were observed in hydrogels, and average pore sizes of the AC_10_A (15% w/v) and AC_10_ARGD (15% w/v) hydrogels were 27.1 ± 7.0 μm and 16.6 ± 6.2 μm, respectively (Fig. 3b). It is possible that the insertion of RGD fragment with positive charge results in the different pore sizes of upper and lower hydrogels at the same concentration.

The color of AC_10_A hydrogel was observed to change from clear to dark green due to the existence of Ag_2_S QDs and Ce6, which verified the successful loading of AD-Ce6/Apt (Fig. 3c). The cells in the MAR hydrogel were stained by calcein AM and propidium iodide (PI) after encapsulation for 2 h, and the strong green fluorescence in 3D confocal image indicated the successful encapsulation and good cellular activity of BMSCs. This result implied that nutrition and oxygen could be transported in the AC_10_ARGD hydrogels due to the proper permeability (Fig. 3d). The AA-MAR hydrogels were obtained by self-healing of AA and MAR hydrogels, which arrived from the non-covalent bonding of same leucine zipper A domain. AC_10_A and AC_10_ARGD hydrogels were placed together for 5 min and the attachment site was recorded. The boundary of AC_10_A and AC_10_ARGD hydrogels was blurred by the self-assembly of A domain (Fig. 3e). This bottom-up assembly process formed a complete and continuous hydrogel from two independent hydrogels (AC_10_A hydrogel and AC_10_ARGD hydrogel).

To evaluate the biocompatibility of hydrogel, the cell viability and the osteogenic differentiation of BMSCs encapsulated in AC_10_ARGD hydrogels were evaluated through the MTT method and alkaline phosphatase (ALP) staining. The obvious increase of cell survival rate (77.1% to 91%) was observed after encapsulating by AC_10_ARGD hydrogels, pointing that AC_10_ARGD hydrogels provided a protective barrier to BMSCs as co-cultured with AD-Ce6/Apt (Additional file 1: Fig. S3). No difference in the ALP expression was found between the MAR group and PBS group (Additional file 1: Fig. S4), indicating that BMSCs were not affected on the ability of osteogenesis by encapsulation within hydrogel.

### Release kinetics of AA and AA-MAR hydrogels

Previous studies showed that AC_10_A hydrogel presented the rapid erosion and sequential release in open solution because AC_10_A has a strong tendency to form intro-molecular loops [40]. To assess the AD-Ce6/Apt release kinetics, release behavior curves of the 2-mm-thick AA hydrogel (AC_10_A: 15% w/v, AD-Ce6: 4 μg\mL) in ultrapure water were recorded (Fig. 3f). The results exhibited that the initial burst release (68.9%) of AD-Ce6/Apt from AA hydrogel was occurred within the first 180 min, and the release rate became constant in 240 min. While only 20.89% of Ce6 from the AC_10_A hydrogel loaded with Ce6 was released within 480 min. This was probably due to the fact that the higher hydrophilia of Ce6 graft-modified with phospholipid contributed to quick release in the open water solution, while hydrophobic free Ce6 was stuck in the hydrophobic cavities of AC_10_A hydrogels.

The sequential release of AA-MAR hydrogel was investigated in PBS and recorded at 0, 4, 8, 12 h. The obvious bilayer structure of AA-MAR hydrogel was exhibited at 0 h. The distinctly sequential release can be observed with the swelling of AA-MAR hydrogel at 4 and 8 h. And the AA-MAR hydrogels were completely swollen and released at 12 h (Fig. 3g). These results confirmed that this self-assembled bilayer hydrogel can realize the rapid and sequential release in PBS.

### In vitro PDT efficiency of AA hydrogel against bacteria

**Fig. 4:**
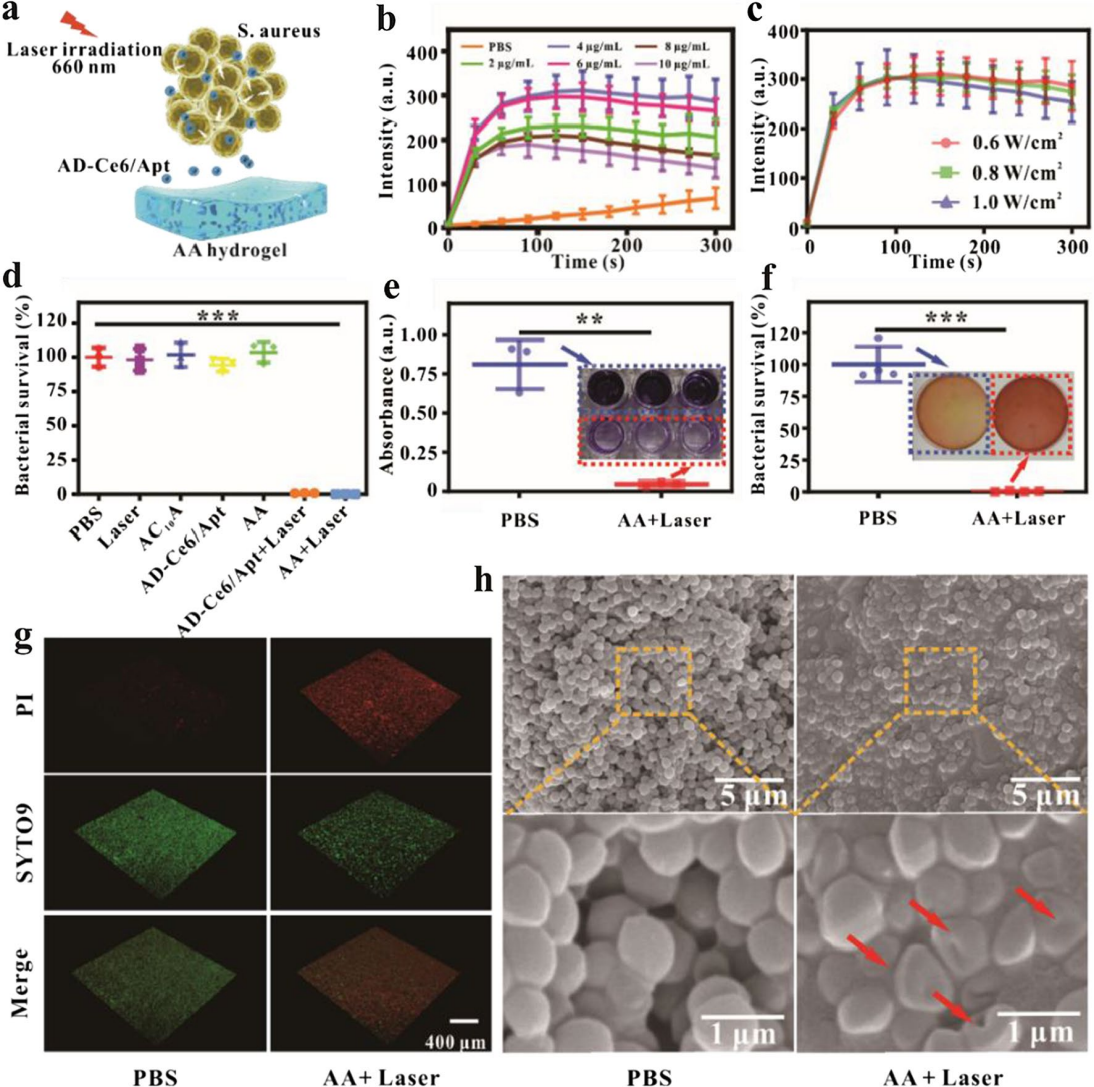
In vitro antibacterial property of AA hydrogels. **a** Schematic illustration of antibacterial procedure. Fluorescence intensity of SOSG after adding with different concentrations of AD-Ce6/Apt (**b**) and irradiation with a laser of different power densities (**c**). **d** Survival rates of *S. aureus* after different treatments (***p < 0.001, n = 5). **e** Macroscopic crystal violet stained images and corresponding absorbance values of *S. aureus* incubated with AA hydrogels for 48 h and irradiated with a laser (660 nm, 0.6 W cm^−2^) for 5 min (**p < 0.01, n = 5). **f** Typical photographs and counting results of *S. aureus* biofilms treated with PBS and AA + Laser groups by the SPM (***p < 0.001, n = 5). **g** 3D-reconstructions of the live/dead stained biofilms of *S. aureus* treated with PBS and AA + Laser. **h** High-resolution SEM images of *S. aureus* biofilm treated with PBS and AA + Laser

^1^O_2_ produced by AD-Ce6/Apt can induce oxidative damage of biomacromolecules (protein, lipid, and DNA) and metabolic dysfunction of bacteria. Therefore, photodynamic performance of AD-Ce6/Apt determines the sterilization efficiency (Fig. 4a). To investigate the photodynamic performance of AD-Ce6/Apt under 660 nm laser irradiation, singlet oxygen sensor green (SOSG) was used to detect the ^1^O_2_ production, and the fluorescence intensity was recorded every 30 s. The PDT efficiency of AD-Ce6/Apt with different concentrations (2, 4, 6, 8, and 10 μg\mL) against bacteria was first measured. The fluorescence intensity of SOSG first increased with the increasing of AD-Ce6/Apt concentrations (from 2 to 4 μg\mL), reaching the maximum at the AD-Ce6/Apt concentration of 4 μg\mL, and then gradually decreased with the increasing of AD-Ce6/Apt concentrations. It is probably due to the aggregation-caused quenching of Ce6 at high concentration of AD-Ce6/Apt. Therefore, AD-Ce6/Apt of 4 μg\mL with best photodynamic performance was chosen for anti-bacteria. Considering the effect of power density on photodynamic performance, the fluorescence intensity of SOSG in the presence of 4 μg\mL AD-Ce6/Apt under 660 nm laser irradiation of different power densities (0.6, 0.8, 1.0 W cm^−2^, 5 min) was also assessed (Fig. 4c). The results showed that no distinct difference of the fluorescence intensity was found. Thus, to avoid the damage of high power density to the skin, a power density of 0.6 W cm^−2^ was used in the following research.

Biofilm formed by colonizing bacteria is the main reason for the stagnation of osteomyelitis clinical treatment effect and results in the immunosuppression and drug resistance [2, 41]^.^ The abilities of AA hydrogel loaded with AD-Ce6/Apt to remove *S. aureus*, inhibit biofilm formation, and eliminate biofilm were further investigated. To assess *S. aureus* elimination rate of AA hydrogel under 660 nm irradiation (0.6 W cm^−2^, 5 min), bacteria suspension solutions (OD_600_ = 0.1, 10^8^ CFU\mL) cultured in 96-wells were divided into seven groups and subjected from different treatments, including PBS, AC_10_A hydrogel, AD-Ce6/Apt, AA, Laser, AD-Ce6/Apt + Laser, and AA + Laser. There was little change of the bacterial survival rates in the AC_10_A hydrogel, AD-Ce6/Apt, AA, and Laser groups compared with the PBS group, indicating that individual AC_10_A hydrogel, AD-Ce6/Apt, AA, or laser was no toxicity to *S. aureus*. In contrast, the bacterial survival rates of AD-Ce6/Apt + Laser and AA + Laser groups decreased rapidly (n = 5, ***p < 0.001) (Fig. 4d). These results suggested that ^1^O_2_ produced by AD-Ce6/Apt in the AA hydrogel under laser irradiation resulted in outstanding antibacterial effect. And the negligible difference of bacterial survival rates in AD-Ce6/Apt + Laser and AA + Laser groups suggested that the sterilizing performance of the AD-Ce6/Apt was no discernible difference before and after release. Simultaneously, the bacterial suspensions after different treatments were cultured for 48 h, and the formation of biofilm was observed by crystal violet staining. Compared with the control groups, AA hydrogel + Laser group exhibited very weak crystal violet staining (Fig. 4e). To investigate the capacity of AA hydrogel to remove mature biofilm, bacteria suspension solutions limpidly cultured in 24-wells for 2 days, and the efficiency of anti-biofilm was investigated through counting bacterial colonies by SPM after different treatments. The bacterial survival rate of AA hydrogel + Laser group was much lower than those of PBS groups (n = 5, ***p < 0.001), indicating that the excellent photodynamic performance of AD-Ce6/Apt inside AA hydrogel resulted in good killing effect even on mature biofilm (Fig. 4f). The efficiency of anti-biofilm was also measured by live/dead staining assay. As shown in Fig. 4g, few red-stained area can be seen in the PBS groups, and a mass of red region can be found in the AA hydrogel + Laser group, which was consistent with the SPM results. This result indicated that the vast damage of *S. aureus* cell wall and membrane in the AA hydrogel + Laser group. In addition, the morphology of biofilm after different treatments was observed by SEM. The most *S. aureus* lost its origin sphere-like morphology in the AA hydrogel + Laser group with cracked biofilms, and more bacteria maintained a complete sphere in the PBS groups (Fig. 4h). These results demonstrated the excellent ability of AA hydrogel to eliminate biofilm.

### In vitro immunomodulation assay of macrophages

The cytotoxicity of AD-Ce6/Apt within AA-MAR hydrogel was an important indicator of whether it could be used in clinical experiments. The MTT assay was used to investigate the cytotoxicity of AD-Ce6/Apt in vitro. The high cell viabilities (94.8% for NIH 3T3, 97.8% for HUVEC, and 97.8% for rat bone marrow-derived macrophages (BMDMs)) were found after incubation with AD-Ce6/Apt at the Ce6 concentration of 4 μg\mL for 72 h, indicating that the AD-Ce6/Apt was low toxic to cells (Additional file 1: Fig. S5).

**Fig. 5:**
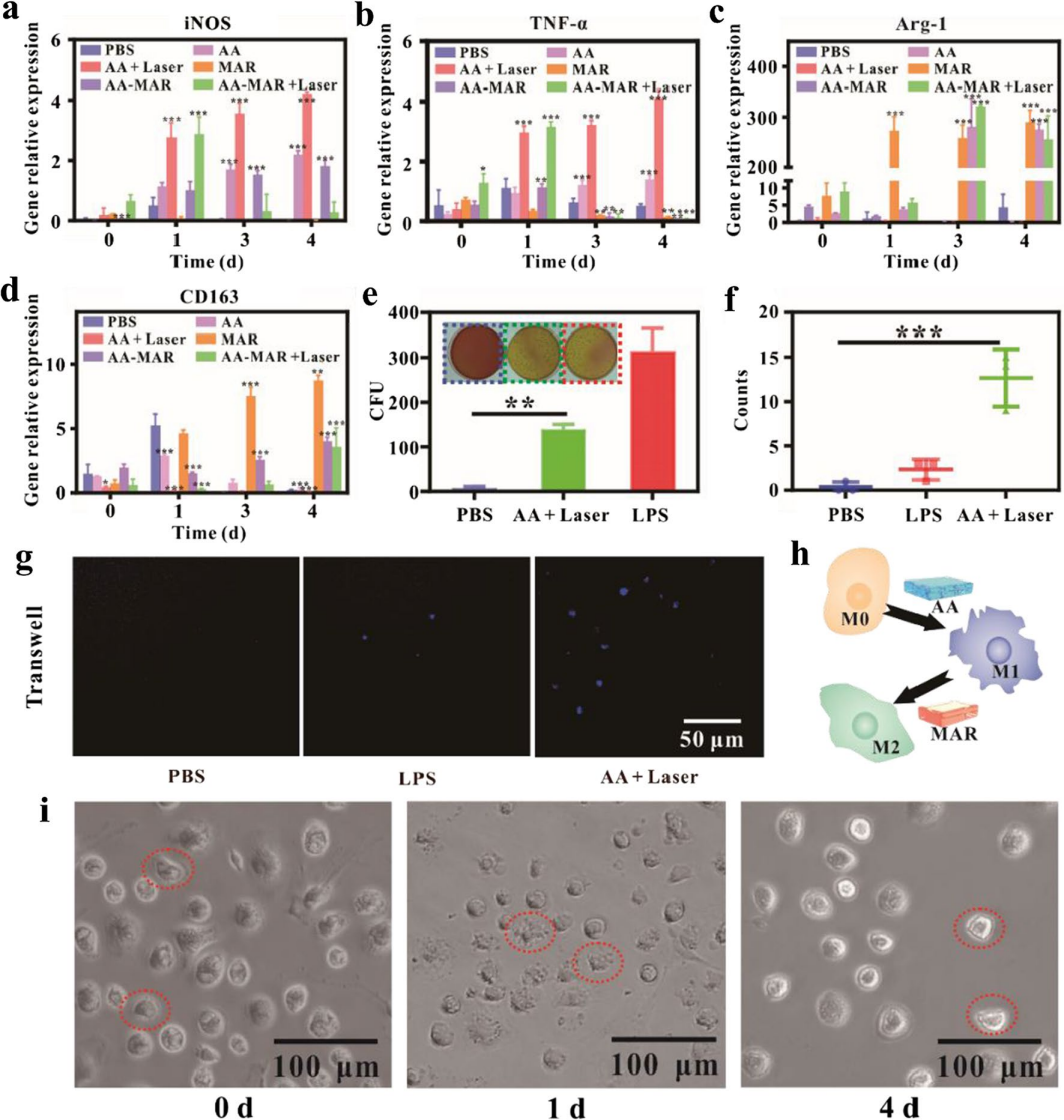
Effects of AA-MAR hydrogel on BMDMs. Real-time PCR of M1&M2 markers and inflammatory cyotokines **a** iNOS, **b** TNF-α, **c** Arg-1, and **d** CD163 expression of BMDMs after stimulated by PBS or AA-MAR for 0, 1, 3, and 4 days (*p < 0.05, **p < 0.01, ***p < 0.001, and ****p < 0.0001, n = 5). **e** Photograph and quantification of *S. aureus* phagocytized by BMDMs after treatment with PBS, LPS, or AA + Laser. **f** The quantitative results of BMDMs. **g** Images of BMDMs cultured on transwells and stimulated by PBS, LPS, or AA + Laser. BMDMs were stained with DAPI. **h** Schematic illustration of morphology transformation of macrophage following the polarization process. **i** Morphological changes of BMDMs during differentiation after stimulated with AA-MAR + Laser on day 0, 1, and 4

Considering that ^1^O_2_ generated by AD-Ce6/Apt could clear bacteria effectively, there was still a risk of residual bacterial and M2 type macrophages could be induced to suppress local nonspecific immunity once forming the biofilm [3, 42] To address the issue, AD-Ce6/Apt in the AA hydrogel was designed to recruit and activate macrophages reprogramming toward M1 phenotype through generating extracellular ROS, which further eliminated pathogens in infected tissues and enhance antibacterial efficiency [7, 8]. Real-time quantitative polymerase chain reaction (RT-qPCR) was employed to evaluate the macrophage phenotype at a gene level (Fig. 5a–d). BMDMs were divided into three groups (PBS, AA, and AA + Laser), and the expression of tumor necrosis factor (TNF-α), inductively nitric oxide synthase (iNOS, proinflammtory cytokine), arginine (Arg-1), and cluster of differentiation 163 (CD163, anti-inflammatory cytokine) were examined after different treatments for 4 days. AA + Laser exhibited the most powerful ability to activate macrophage reprogramming toward M1 phenotype with significant increase of pro-inflammatory gene (TNF-α and iNOS). Although a persistent elevation of TNF-α and iNOS expression was observed in the AA group, the ability of triggering macrophage polarization was obviously limited compared with the AA + Laser group. This result indicated that ROS produced by the AA hydrogel under laser irradiation possessed more powerful inductivity, while AA hydrogel as alien invaders can induce macrophages to exhibit M1 phenotype. Simultaneously, the phagocytic ability of triggered M1-like macrophage in AA + Laser group was assessed. Phagocytized bacteria were collected by splitting macrophages and counted by the SPM. The colony-forming unit (CFU) of the AA + Laser groups was much higher than that of PBS groups, showing the phagocytic ability of triggered M1-like macrophage in the AA + Laser groups (Fig. 5e). While the CFU of LPS groups (100 ng\mL) was higher than that of AA + Laser groups, suggesting that the phagocytic ability of triggered M1-like macrophage in the AA + Laser groups was inferior to that in the LPS groups. To measure the effect of microenvironment on macrophage migration, BMDMs were cultured with PBS, LPS, or AA + Laser in the transwell, and cells migrating to the bottom of wells were counted. After 24 h of culture, cells in PBS groups persisted in quiescent and inactive state, remaining at their original location. The addition of LPS caused few cells migrating to the bottom. Cells exposed to AA hydrogel were greatly enhanced to migration under laser irradiation (n = 5, ***p < 0.001) (Fig. 5f, g). Thus, the presence of AA + Laser group was distinct associated with an enhancement of BMDMs migration, which looked like the result of BMDMs activation by ROS. These results proved that the AA hydrogel could not only promote inflammation, but also recruit macrophage to further eliminate pathogens. It was expected to be a good immunomodulatory reagent to reverse immunosuppression around infected site and resisted the attack of bacteria in vivo.

After clearing away the pathogens in the first stage, inflammation stage must be changed to anti-inflammation state to promote osteogenesis. The well osteogenesis repairment was taken into account, and the MAR hydrogel layer was designed for timely induce macrophage to perform M2 phenotype, which assisted in building the osteo-immune environment to promote bone reconstruction [42, 43]. To confirm the function of the MAR hydrogel in vitro, BMDMs were cultured with PBS or MAR hydrogel for 4 days and then collected for RT-qPCR analysis. Compared with the PBS group, the expression of anti-inflammation cytokines (Arg-1, CD163) was obviously promoted in the MAR groups (Fig. 5a–d), indicating that independent MAR suppressed the inflammation.

To investigate whether the AA-MAR hydrogel can approach same immunoresponse through sequential release in vitro, the primary BMDMs was co-cultured with AA-MAR for 4 days. Compared with the negative PBS group, AA-MAR obviously promoted inflammation after co-culturing for 1 day, and TNF-α and iNOS expressions were prominent up-regulated. While TNF-α and iNOS expressions decreased gradually, and the expressions of anti-inflammation genes (Arg-1 and CD163) were promoted on day 3 and 4 (n = 5, *p < 0.05, **p < 0.01, ***p < 0.001) (Fig. 5a–d). These results indicated that the AA-MAR hydrogel can regulate the spatiotemporal transformation of macrophages phenotype through sequential release (Fig. 5h). Consistent results were observed in the morphology of BMDMs under bright field microscope. The morphology of macrophages treated with AA-MAR + Laser displayed either “fried-egg” or “elongated” shapes with numerous filopodia extensions compared with the PBS group on d 1. After treatment with AA-MAR for 4 days, the adherent macrophages presented “spindle-like” or “round” shapes (Fig. 5i). Previous researches showed that BMDMs with spindle-like morphology facilitated tissue and wound repair and had anti-inflammation phenotype (M2 phenotype), and with “fried-egg” morphology exhibited pro-inflammation behavior (M1 phenotype) [44]. The morphological data clearly presented that the BMDMs in the AA-MAR hydrogel can be phenotypically transformed from M1 to M2 after laser irradiation. These results confirmed that the AA-MAR + Laser can realize the immune microenvironment transformation by regulating macrophage phenotype in vitro.

### The anti-bacterial effects of AA-MAR hydrogel in vivo

**Fig. 6:**
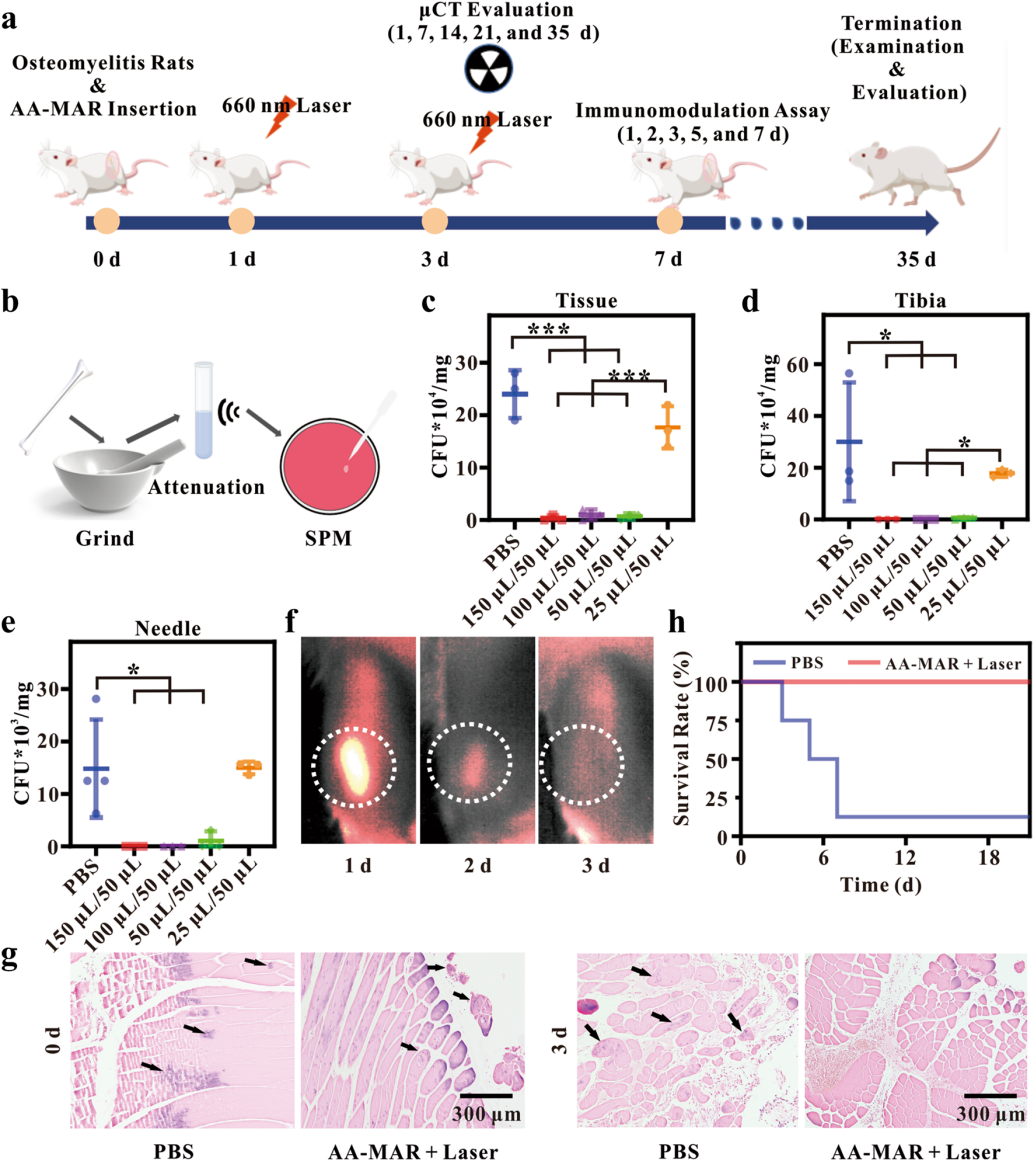
Evaluation of AA-MAR for in vivo treatments and anti-infection. **a** Scheme of treatment, examination, and evaluation of S. aureus induced osteomyelitis rats at different time points. **b** The processing steps of tibias, tissues, and implants. Quantification of S. aureus collected from the tissue (**c**), tibia (**d**), and needle (**e**) on day 3 post-fracture by SPM after treatment with different proportions of AA-MAR + Laser (*p < 0.05, **p < 0.01, and ***p < 0.001, n = 5). **f** In vivo NIR fluorescence imaging of rats after subcutaneously injected with AA-MAR. **g** Histological section of tibias post-fracture from Gram staining in different groups on day 0 and 3. **h** Overall survival rate of S. aureus induced osteomyelitis rats treated with PBS or AA-MAR + Laser

The encouraging anti-infection and immunomodulatory effects of AA-MAR hydrogel stimulated it to cure osteomyelitis in vivo. Considering the possibility of unnecessary damage from excessive ^1^O_2_ generated by the AA-MAR to normal tissue, a series of volume ratio of the AA-MAR hydrogel (150 μL/50 μL, 100 μL/50 μL, 50 μL/50 μL, and 25 μL/50 μL) were preliminary optimized to avoid adverse side effects. The different proportions of AA-MAR hydrogels were implanted in the fractured tibia of *S. aureus*-induced osteomyelitis rats (Fig. 6a). The right tibias of *S. aureus*-induced osteomyelitis rats treated with the AA-MAR hydrogel were exposed to 660 nm irradiation (0.6 W cm^−2^, 5 min) on d 1 and d 3, and the efficiency of anti-bacteria was assessed by collecting the wounded tissues, tibia, and needles for bacterial culture on d 3. To visualize the results, the collected samples were treated with ultrasonic concussion and quantified by the SPM (Fig. 6b and Additional file 1: Fig. S6). Needles, tissues, and bones in 25 μL/50 μL group displayed a large amount of colonies, and quantification results have no significant difference with the PBS group (Fig. 6c–e). When the volume ratio increased over 25 μL/50 μL, the colony-forming unit counts of needles, tissues, and bones in 150 μL/50 μL, 100 μL/50 μL, and 50 μL/50 μL groups had a significant decrease compared with those in blank and 25 μL/50 μL groups (n = 5, *p < 0.05, ***p < 0.001). To avoid side effect causing by excessive ^1^O_2_ produced by the AA hydrogel, 50 μL/50 μL of AA-MAR hydrogel with excellent antibacterial effect was selected for subsequent investigation.

It was noteworthy that the AA hydrogel was needed to fast degrade within 3 days to eliminate bacteria and avoid biofilm formation in case of repeated infection and immune suppression [44]. AA-MAR hydrogels (50 μL/50 μL) were injected subcutaneously, and the degradation of AA hydrogel was monitored by observing the fluorescence intensity of Ag_2_S QDs in the AD-Ce6/Apt (Fig. 6f). The fluorescence intensity of Ag_2_S QDs faded over time and disappeared on day 3, indicating the complete degradation of the upper AA hydrogel within 3 days in vivo.

To further investigate the antibacterial effect with the degradation of the upper AA hydrogel, Gram staining of *S. aureus*-induced osteomyelitis rat tissues were conducted and analyzed at 0 and 3 days after treating with AA-MAR hydrogel (50 μL/50 μL) and laser irradiation (Fig. 6g). Owing to establishment of *S. aureus* induced osteomyelitis rats, a large number of viable bacteria (black arrows) can be found from infected tissues of all groups on day 0. More bacteria can be found with the aggravation of tissue infection degree in the PBS group on day 3, which invaded and spread more widely than that of on day 0. In contrast, few *S. aureus* can be observed in the AA-MAR + Laser group, indicating that photodynamic treatment of the AA-MAR contributed to anti-bacteria in the infected tissues of *S. aureus* induced osteomyelitis rats. Simultaneously, the life span of *S. aureus* induced osteomyelitis rats was recorded to evaluate the survival rate from modeling to 5 weeks postsurgery. It was found that prompt treatment with AA-MAR + Laser can increase the survival rate to 100%. Due to rapid invasion and proliferation of pathogens, the survival rate of untreated S. aureus induced osteomyelitis rats was found to quickly decrease to 12.5% after infection for 7 days, while the life span of S. aureus induced osteomyelitis rats in the AA-MAR + Laser group was significantly prolonged (Fig. 6h). These results demonstrated that the AA-MAR hydrogel exhibited superior antibacterial efficiency of osteomyelitis treatment with the degradation of the AA hydrogel, which played an important role to prolong the life span of S. aureus induced osteomyelitis rats.

### Immunoresponse induced with AA-MAR hydrogel in vivo

**Fig. 7:**
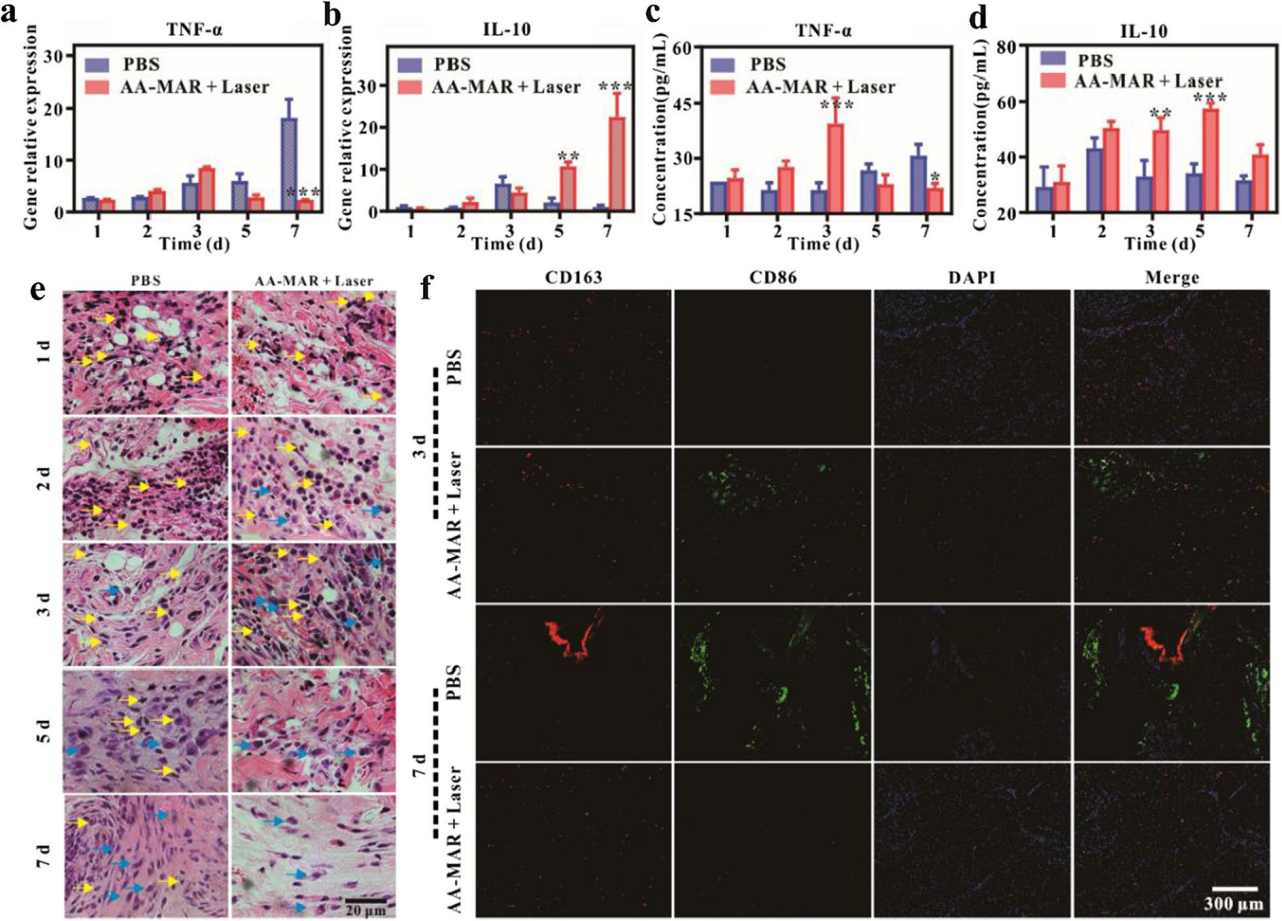
In vivo immune responses of S. aureus-induced osteomyelitis rats treated with AA-MAR. Real-time PCR of **a** TNF-α and **b** IL-10 around infected area on day 1, 2, 3, 5, and 7. Elisa results of **c** TNF-α and **d** IL-10 in serum on day 1, 2, 3, 5, and 7 (*p < 0.05, **p < 0.01, and ***p < 0.001, n = 5). **e** Hematoxylin and eosin staining results of tibias post-fracture after treatment with PBS or AA-MAR + Laser for 1, 2, 3, 5, and 7 days (yellow arrows indicate neutrophils; blue arrows indicate macrophages). **f** Representative immunofluorescence images of CD86 (green) and CD163 (red) in tissue samples after treatment with PBS and AA-MAR + Laser for 3 and 7 days

After the disruption of pathogens, the anti-inflammatory efficiency of the AA-MAR hydrogel during bone infection was examined in different time points. To validate the immune microenvironment spatial–temporal transformation triggered by the sequential release of the AA-MAR hydrogel, the expression of relevant cytokines was assessed at gene and protein levels. Local tissue samples around the infected sites were gathered to investigate the expression of inflammation cytokine (TNF-α) and anti-inflammation cytokine (IL-10) at 1, 2, 3, 5, and 7 days by RT-qPCR (Fig. 7a, b). The TNF-α expression in local infected tissue of the PBS group increased continuously during the observation period, while that in the AA-MAR + Laser group exhibited uptrend in the first 3 days and then descent until 7 days. The concentration of TNF-α in the AA-MAR + Laser group was significantly lower than that in the PBS group. And the concentration of IL-10 in the AA-MAR + Laser group continued to increase. Simultaneously, serum samples of S. aureus induced osteomyelitis rats were collected to assay the concentration of TNF-α and IL-10 at 1, 2, 3, 5, and 7 days through enzyme-linked immunosorbent assay (Elisa) (Fig. 7c, d). The concentration of TNF-α in the AA-MAR + Laser group climbed steadily in the first 3 days, which was much higher than that in the PBS group on day 3. The concentration of TNF-α in the AA-MAR + Laser group subsequently declined until 7 days, which was lower than that in the PBS group on day 7. As for the concentration of IL-10, the AA-MAR + Laser group exhibited uptrend during the first 5 days. The concentration of IL-10 in the AA-MAR + Laser group was also found significantly different from that in the PBS group on day 3 and 5. While the concentration of IL-10 in the AA-MAR + Laser group declined on day 7, which was attributed to the fact that AA-MAR + Laser did not cause immune cell unrestricted expansion in vivo leading to cytokine storms or cytokine release syndrome (CRS). These results indicated that AA-MAR + Laser promoted inflammation in the first 3 days and inhibited inflammation subsequently in local tissue, which coincided with the sequential degradation time of AA-MAR hydrogel in vivo. It can be reasonable to speculate that an immune environment transformation from pro-inflammation to anti-inflammation was regulated around infected site of fracture without systemic adverse reactions with the sequential release of the AA-MAR hydrogel.

Hematoxylin and eosin (H&E) and immunofluorescence staining of tissue around infected sites were following used to evaluate immunoenvironment transformation after treatment with AA-MAR + Laser. A large number of inflammatory cells were observed in the AA-MAR + Laser group and PBS group on day 3, and the inflammation cells in the AA-MAR + Laser group were much more than that in the PBS group (Additional file 1: Fig. S6). After treatment for 7 days, few inflammatory cells emerged from the AA-MAR + Laser group, while the degree of inflammatory infiltration was exacerbated in the PBS group. The quantity of neutrophils (yellow arrows) and macrophages (blue arrows) was also counted to assess the degree of inflammation by magnifying H&E stained tissue around infected sites (Fig. 7e and Additional file 1: Fig. S8). Owing to bacterial invasion in fracture sites of osteomyelitis rats, an amount of neutrophils can be observed in PBS and AA-MAR + Laser groups which removing bacteria and foreign object on day 1. And the counts of neutrophils in AA-MAR + Laser groups continued to decline, while that in PBS groups kept elevating, indicating that the prominent antibacterial effect of AA-MAR + Laser alleviated the infiltration of neutrophils, and persistent infection of PBS groups gave rise to the aggravation of inflammation. As to the quantitative results of macrophages, there were much more macrophages can be observed in the AA-MAR + Laser groups than that in PBS groups on day 2 and 3, which attributing to the rapid release of AA hydrogel in AA-MAR + Laser groups within the first 3 days, generated extracellular ROS recruited much more macrophages to local infection sites. And less macrophages in AA-MAR + Laser groups than that in PBS groups on day 7. To further investigate the phenotype of macrophages, immunofluorescence staining of CD86 (M1 marker, inflammation cytokine) and CD163 (M2 marker, anti-inflammation cytokine) was conducted after treatment for 3 and 7 days, and fluorescence intensity ratio of CD86/CD163 (M1/M2) was quantified (Fig. 7f and Additional file 1: Fig. S9). The fluorescence intensity of CD86 in the AA-MAR + Laser group was stronger than that in the PBS group on day 3, and the quantified result of fluorescence intensity ratio of CD86/CD163 in the AA-MAR + Laser group was higher than that in the PBS group. Although the fluorescence intensity of CD163 in the PBS group was much stronger than that in the AA-MAR + Laser group on day 7, the fluorescence intensity ratio of CD86/CD163 in the AA-MAR + Laser group was much lower than that in the PBS group, which are consistent with the results of RT-qPCR, indicating that AA-MAR + Laser can facilitate spatiotemporal immunoenvironment transformation from pro-inflammation to anti-inflammation in vivo.

### Evaluation of fracture healing

**Fig. 8:**
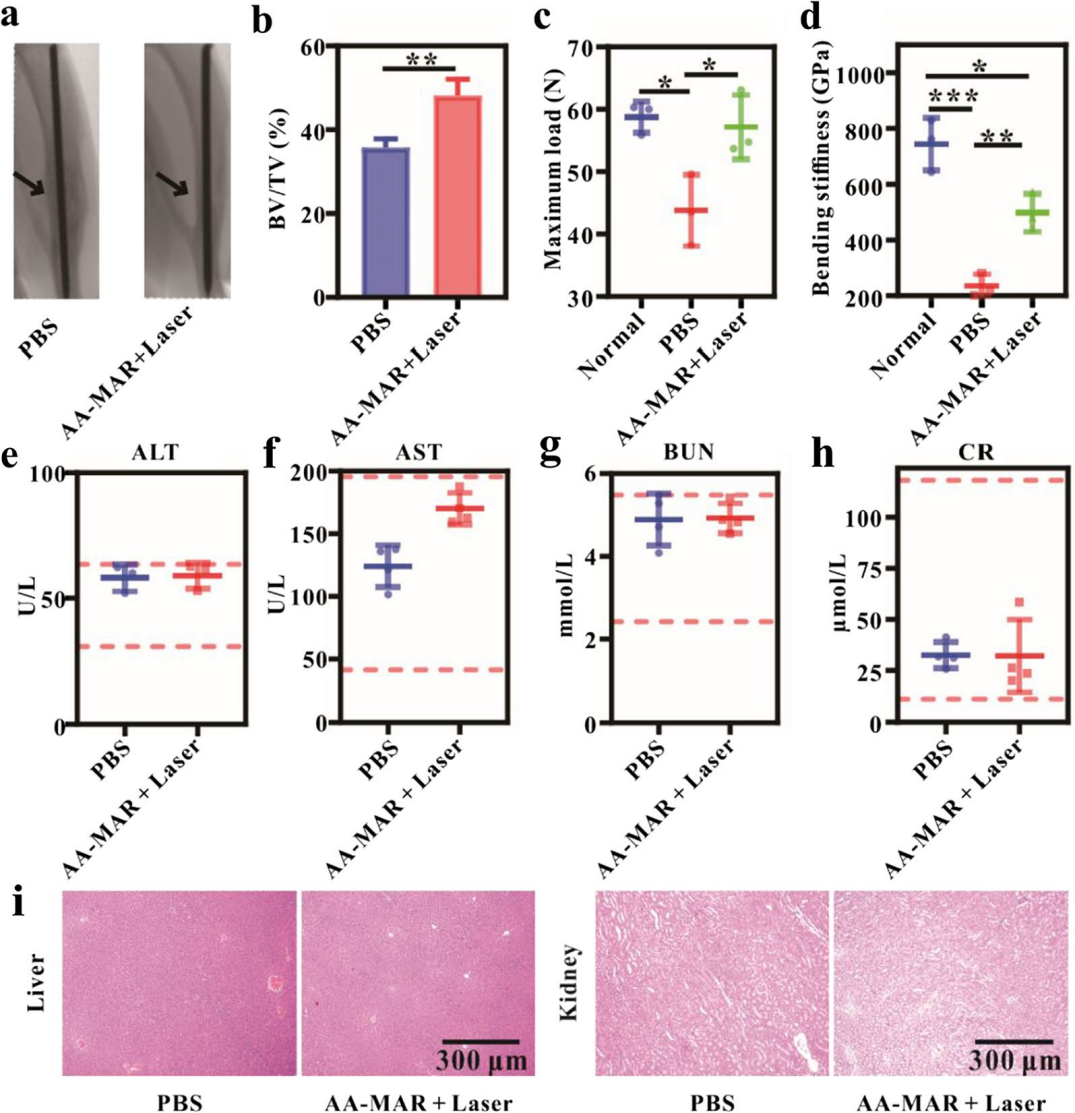
Evaluation of fracture healing. **a** Micro-CT images of tibias from PBS and AA-MAR + Laser groups. **b** Quantitative analysis of bone mass in PBS and AA-MAR + Laser groups (**p < 0.01, n = 5). Biomechanical properties **c** maximum stress, **d** bending stiffness) of tibias were assessed by in vitro three-point bending test (*p < 0.05, **p < 0.01, and ***p < 0.001, n = 5). The serum biochemical assay for **e** ALT, **f** AST, **g** BUN, and **h** CR of *S. aureus*-induced osteomyelitis rats at 5 weeks post-operation. **i** H&E staining of livers and kidneys of *S. aureus*-induced osteomyelitis rats PBS and AA-MAR + Laser groups at 5 weeks post-operation

To determine whether the AA-MAR hydrogel and laser irradiation could effectively promoted bone remodeling in *S. aureus*-induced osteomyelitis rats, we evaluated functional fracture healing at 5 weeks post-operation. Micro-computed tomography was used to analyze the fracture repair and quantify the volume of new bone. As marked by black arrows in Fig. 8a, malunion was found in the survival rates of the PBS group, while the morphology of tibia in the AA-MAR + Laser group presented normal. To analyze the bone mass of the AA-MAR + Laser group and PBS group, we found that the bone volume/tissue volume (BV/TV) ratio of positive group (48.16%) was much higher than those of negative group (35.79%) (n = 5, **p < 0.01) (Fig. 8b). Importantly, three-point bending testing was also used to investigate the mechanical integrity of the tibia. The maximum load and bending stuffiness of infected fractures treated with the AA-MAR hydrogel were significantly higher than that of the PBS group (n = 5, *p < 0.05, **p < 0.01, ***p < 0.001) (Fig. 8c, d), suggesting that osteogensis would be inhibited by intensely competition of osteoblast on the surface of implant during osteomyelitis. These results demonstrated that the AA-MAR hydrogel leaded to efficient healing by providing a reliable and timely microenvironment conversion.

To assess the potential systemic toxicity of the AA-MAR hydrogel, liver and kidney histology and serum tests were performed at 5 week post-fracture. All values within normal range, and no significant necrosis of liver and kidney cells and abnormal tissue structure were observed in histologic sections (Fig. 8e–i), confirming the safety of the AA-MAR + Laser therapy.

## Conclusion

In conclusion, we developed a bilayer multifunctional AA-MAR hydrogel based on engineered polypeptides AC_10_A and AC_10_ARGD for spatiotemporal modulation of efficiently bactericidal and anti-inflammation process in osteomyelitis treatment. The AD-Ce6/Apt released from AA hydrogel can generate the amount of ^1^O_2_ to kill *S. aureus* and recruit macrophage to the infected site, leading to activation of the M1 phenotype immune system in early stage. Sequentially, BMSCs are released from the MAR hydrogel and differentiate to rebuild fractured bone, allowing spatiotemporal regulation of the immune environment for anti-inflammation and further avoiding side effects caused by excessive inflammation. We believe that the concept of spatiotemporal modulation arrived from self-assembled bilayer hydrogels could be applied as a novel and robust approach to guide the design of multifunctional biomedical materials and achieve the ultimate goal of osteomyelitis treatment.

## Supplementary Information

**Additional file 1:** **Fig. S1.** Schematic representation of pQE9AC_10_A plasmid.** Fig. S2. **Schematic representation of AC_10_A and AC_10_ARGD proteins and the amino acid sequences of major domains. **Table S1.** Primer sequences for real-time PCR analysis. **Fig. S3.** Cell viabilities of BMSCs and BMSCs inside the MAR hydrogel treated with PBS or AD-Ce6/Apt (**p<0.01, ***p ˂ 0.001, n = 5). **Fig. S4.** ALP staining images of BMSCs after culture with PBS or AA-MAR hydrogel in the osteogenic medium for 21 days. **Fig. S5.** Cell viabilities of NIH 3T3, BMDMs, and HUVEC after incubation with AD-Ce6/Apt (4 μg mL^-1^). **Fig. S6. **Typical photographs of bacterial colony from infected tissue, needle, and tibia treated with different proportion of AA-MAR + Laser on S. aureus induced osteomyelitis rats. **Fig. S7. **H&E staining results of tibias post-fracture after treatment with PBS or AA-MAR + Laser for 1, 2, 3, 5, and 7 days. **Fig. S8. **The quantitative results of neutrophils and macrophages (*p < 0.05, **p < 0.01, ***p < 0.001, and ****p < 0.0001, n = 5). **Fig. S9.** The ratio of M1/M2 in different groups on day 3 and 7 (***p ˂ 0.001, n = 5).

## Data Availability

All data generated or analyzed during this study are included in this published article (and its additional files).

## Abbreviations

Ce6: Chlorin e6
Ag_2_S QDs: Ag_2_S quantum dots
AD-Ce6/Apt: Ag_2_S QDs@DSPE-mPEG_2000_-Ce6/Aptamer
AA: AC_10_A hydrogel loading with AD-Ce6/Apt
BMSCs: Bone marrow-derived mesenchymal stem cells
MAR: AC_10_ARGD hydrogel loading with BMSCs
*S. aureus*: *Staphylococcus aureus*
Lps: Lipopolysaccharide
PDT: Photodynamic therapy
PS: Photosensitizers
ROS: Reactive oxygen species
TEM: Transmission electron microscope
SEM: Scanning electron microscope
ALP: Alkaline phosphatase
SOSG: Singlet oxygen sensor green
SPM: Spread plate method
BMDMs: Bone marrow-derived macrophages
RT-qPCR: Real-time quantitative polymerase chain reaction
TNF-α: Tumor necrosis factor
iNOS: Inductively nitric oxide synthase
Arg-1: Arginine-1
CD163: Cluster of differentiation 163
BV/TV: Bone volume/tissue volume
