# Supplementary material for: A self-assembled bilayer polypeptide-engineered hydrogel for spatiotemporal modulation of bactericidal and anti-inflammation process in osteomyelitis treatment
Source: J Nanobiotechnology. 2022 Sep 15;20:416. doi: 10.1186/s12951-022-01614-3 (PMC9479290; doi:10.1186/s12951-022-01614-3)
Supplement: Supplementary file 1 — Additional file 1: Fig. S1. Schematic representation of pQE9AC10A plasmid. Fig. S2. Schematic representation of AC10A and AC10ARGD proteins and the amino acid sequences of major domains. Table S1. Primer sequences for real-time PCR analysis. Fig. S3. Cell viabilities of BMSCs and BMSCs inside the MAR hydrogel treated with PBS or AD-Ce6/Apt (**p<0.01, ***p ˂ 0.001, n = 5). Fig. S4. ALP staining images of BMSCs after culture with PBS or AA-MAR hydrogel in the osteogenic medium for 21 days. Fig. S5. Cell viabilities of NIH 3T3, BMDMs, and HUVEC after incubation with AD-Ce6/Apt (4 μg mL-1). Fig. S6. Typical photographs of bacterial colony from infected tissue, needle, and tibia treated with different proportion of AA-MAR + Laser on S. aureus induced osteomyelitis rats. Fig. S7. H&E staining results of tibias post-fracture after treatment with PBS or AA-MAR + Laser for 1, 2, 3, 5, and 7 days. Fig. S8. The quantitative results of neutrophils and macrophages (*p < 0.05, **p < 0.01, ***p < 0.001, and ****p < 0.0001, n = 5). Fig. S9. The ratio of M1/M2 in different groups on day 3 and 7 (***p ˂ 0.001, n = 5). [file 12951_2022_1614_MOESM1_ESM.docx]

**Additional Information**

**A Self-assembled Bilayer Polypeptide-engineered Hydrogel for Spatiotemporal Modulation of Bactericidal and Anti-inflammation Process in Osteomyelitis Treatment**

Xiaoting Xie,^a1^ Jiemao Wei,^b1^ Bin Zhang,^a^ Wei Xiong,^b*^ Zhiyi He,^b^ Yayun Zhang,^b^ Chenghao, Gao,^b^ Yuandi Zhao,^a,c*^ Bo Liu,^a^

^a^ Britton Chance Center for Biomedical Photonics at Wuhan National Laboratory for Optoelectronics-Hubei Bioinformatics & Molecular Imaging Key Laboratory, Department of Biomedical Engineering, College of Life Science and Technology, Huazhong University of Science and Technology, Wuhan 430074, Hubei, PR China

^b^ Department of Orthopedic Surgery, Tongji Hospital, Tongji Medical College, Huazhong University of Science & Technology, 1095 Jiefang Avenue, Wuhan, Hubei 430030, PR China

^c^ Key Laboratory of Biomedical Photonics (HUST), Ministry of Education, Huazhong University of Science and Technology, Wuhan 430074, Hubei, PR China

^1^ These authors contributed equally to this work.


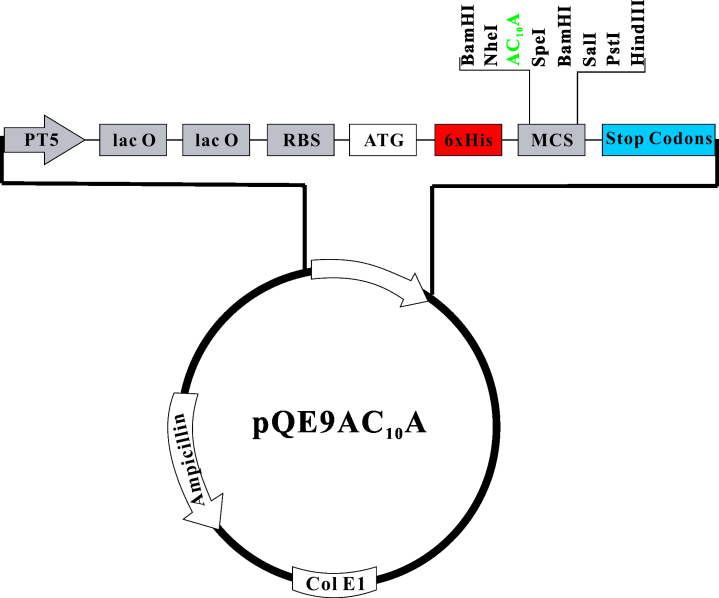


**Fig. S1** Schematic representation of pQE9AC_10_A plasmid.


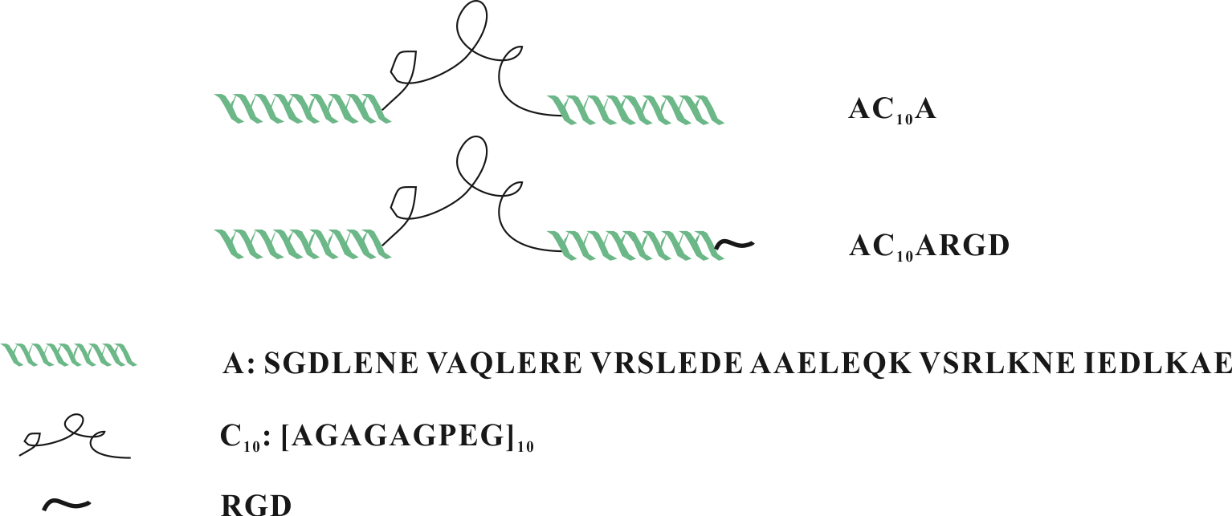


**Fig. S2** Schematic representation of AC_10_A and AC_10_ARGD proteins and the amino acid sequences of major domains.

| Gene | Primer |
| --- | --- |
| GAPDH | Forward: 5’-GGCACAGTCAAGGCTGAGAATG-3’ |
|  | Reverse: 5’-ATGGTGGTGAAGACGCCAGTA-3’ |
| iNOS | Forward: 5’-GAAACTTCTCAGCCACCTTGG-3’ |
|  | Reverse: 5’-CCGTGGGGCTTGTAGTTGAC-3’ |
| TNF-α | Forward: 5’-CCGATTTGCCACTTCATACCA-3’ |
|  | Reverse: 5’-TAGGGCAAGGGCTCTTGATG-3’ |
| Arg-1 | Forward:5’-GCTGTGGTAGCAGAGACCCAGA-3’ |
|  | Reverse:5’-CATCCACCCAAATGACGCATAG-3’ |
| CD163 | Forward:5’-CTGGGATGTCCAACTGCCAT-3’ |
|  | Reverse:5’-AATGCTTCCCCCATTCCTGG-3’ |

**Table S1** Primer sequences for real-time PCR analysis.


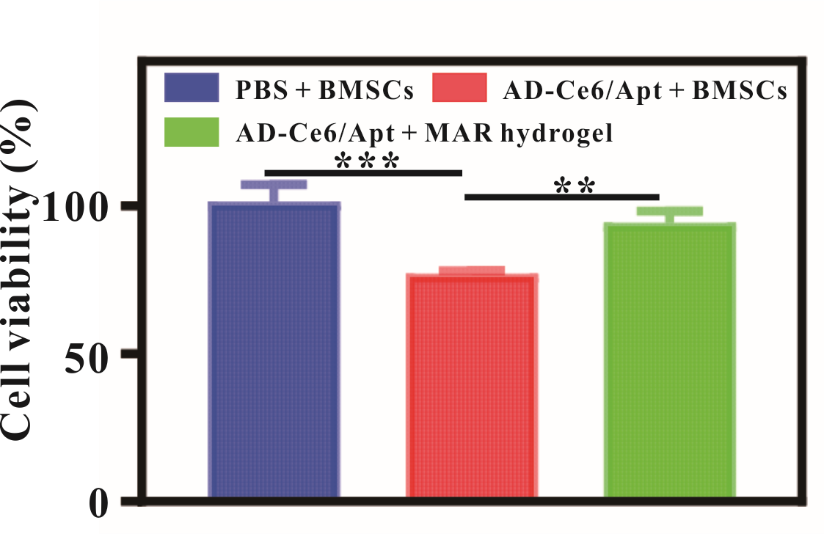


**Fig. S3** Cell viabilities of BMSCs and BMSCs inside the MAR hydrogel treated with PBS or AD-Ce6/Apt (**p<0.01, ***p ˂ 0.001, n = 5).


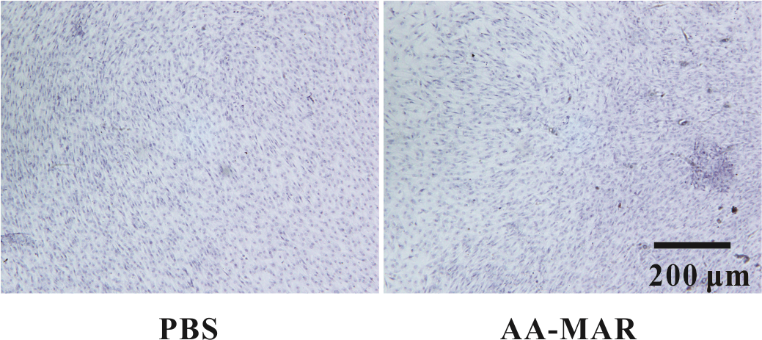


**Fig. S4** ALP staining images of BMSCs after culture with PBS or AA-MAR hydrogel in the osteogenic medium for 21 days.


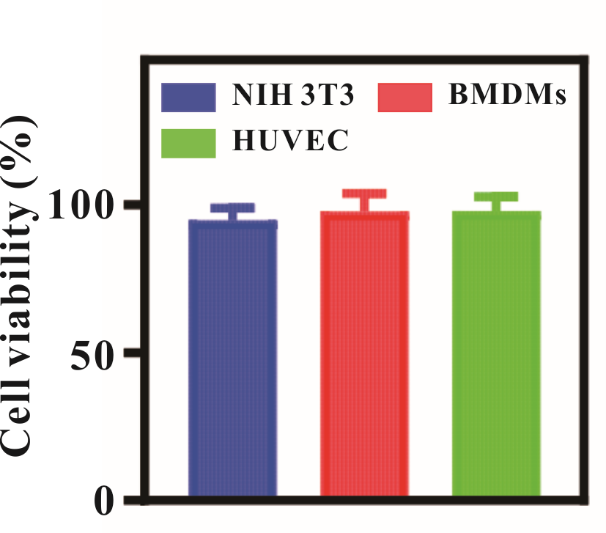


**Fig. S5** Cell viabilities of NIH 3T3, BMDMs, and HUVEC after incubation with AD-Ce6/Apt (4 μg mL^-1^).

**
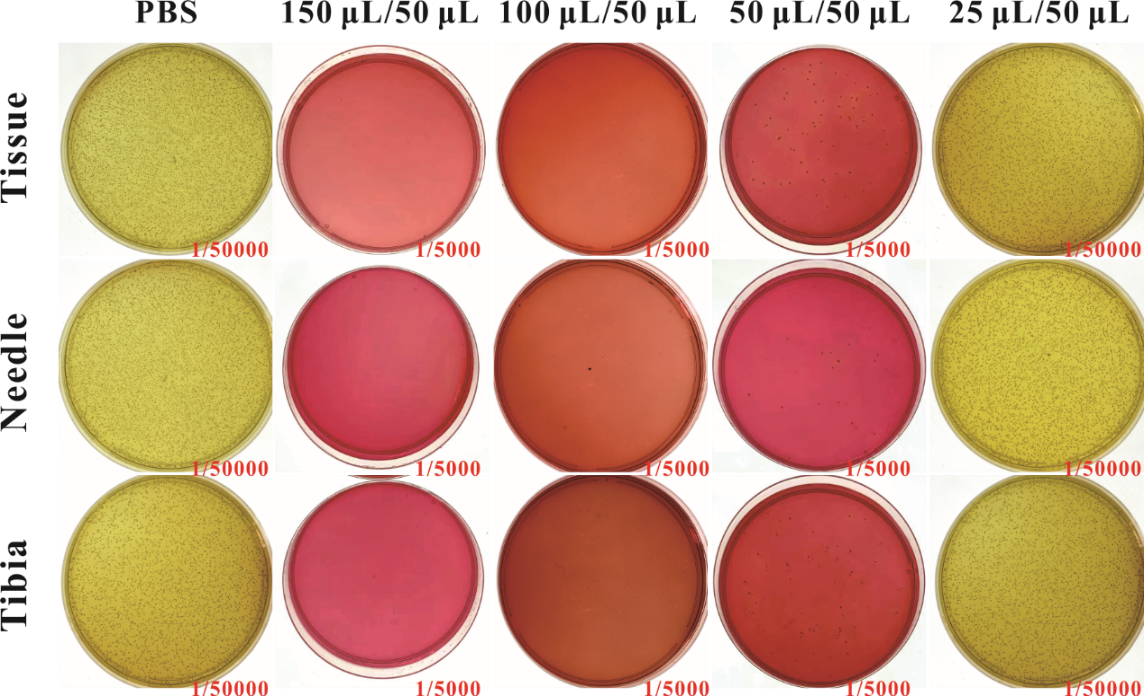
**

**Fig. S6** Typical photographs of bacterial colony from infected tissue, needle, and tibia treated with different proportion of AA-MAR + Laser on S. aureus induced osteomyelitis rats.

**
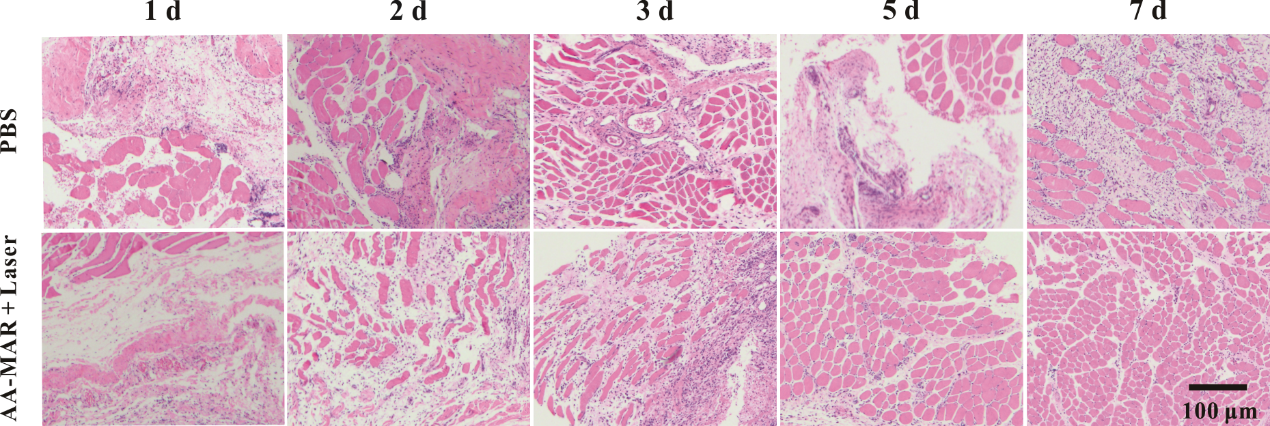
**

**Fig. S7** H&E staining results of tibias post-fracture after treatment with PBS or AA-MAR + Laser for 1, 2, 3, 5, and 7 days.

**
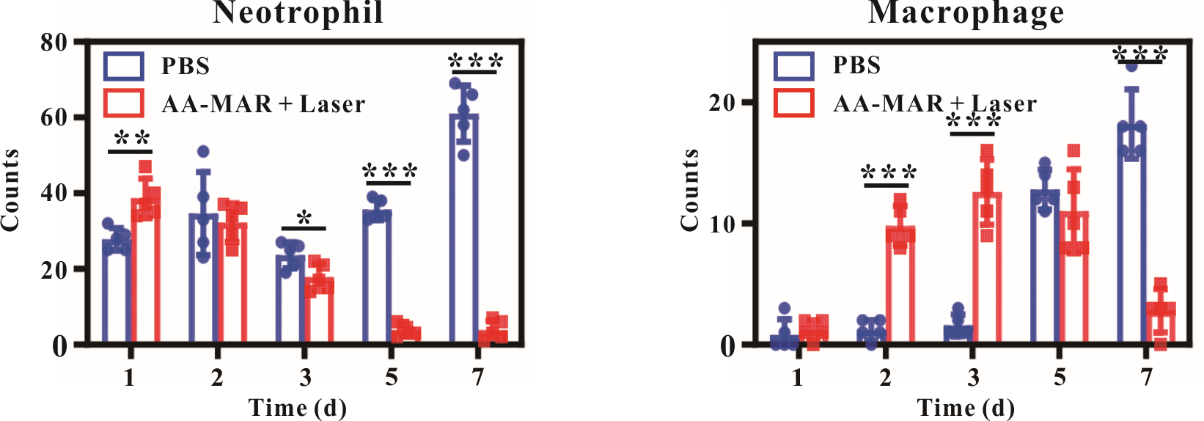
**

**Fig. S8** The quantitative results of neutrophils and macrophages (*p < 0.05, **p < 0.01, ***p < 0.001, and ****p < 0.0001, n = 5).


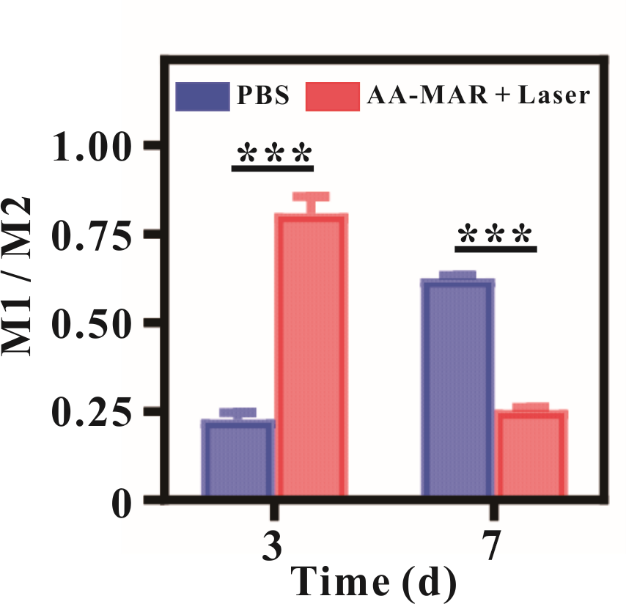


**Fig. S9** The ratio of M1/M2 in different groups on day 3 and 7 (***p ˂ 0.001, n = 5).
